# Sensitivity Analysis of Excess Mortality due to the COVID‐19 Pandemic

**DOI:** 10.1111/padr.12475

**Published:** 2022-03-03

**Authors:** Marília R. Nepomuceno, Ilya Klimkin, Dmitri A. Jdanov, Ainhoa Alustiza‐Galarza, Vladimir M. Shkolnikov

**Keywords:** country comparison, COVID‐19, excess mortality

## Abstract

Estimating excess mortality is challenging. The metric depends on the expected mortality level, which can differ based on given choices, such as the method and the time series length used to estimate the baseline. However, these choices are often arbitrary, and are not subject to any sensitivity analysis. We bring to light the importance of carefully choosing the inputs and methods used to estimate excess mortality. Drawing on data from 26 countries, we investigate how sensitive excess mortality is to the choice of the mortality index, the number of years included in the reference period, the method, and the time unit of the death series. We employ two mortality indices, three reference periods, two data time units, and four methods for estimating the baseline. We show that excess mortality estimates can vary substantially when these factors are changed, and that the largest variations stem from the choice of the mortality index and the method. We also find that the magnitude of the variation in excess mortality is country‐specific, resulting in cross‐country rankings changes. Finally, based on our findings, we provide guidelines for estimating excess mortality.

## Introduction

Excess mortality is one of the most reliable approaches for measuring the impact of the COVID‐19 pandemic. The metric can be estimated by measuring the difference between mortality from all causes that is *observed* during the pandemic and mortality from all causes that would be *expected* if the pandemic had not occurred (baseline mortality). In considering deaths from all causes, excess mortality is independent of the COVID‐19 testing capacity, the definition of COVID‐19 deaths, and the misclassification of COVID‐19 deaths on death certificates (Gill and DeJoseph 2020; Leon et al. 2020). Moreover, the metric includes deaths that are both directly and indirectly attributable to SARS‐CoV‐2 (Ackley et al. 2021). As a result, estimating excess mortality has been considered the best approach for assessing and comparing the overall mortality burden due to the COVID‐19 pandemic across time and space (Beaney et al. 2020).

Despite its advantages, estimating excess mortality is not a trivial task. Excess mortality estimates depend on the baseline, which can vary depending on the mortality index (death counts or rates), the number of years included in the reference period, and the method used to estimate the baseline (Németh, Jdanov, and Shkolnikov 2021; Schöley 2021). In addition, excess mortality estimates may change depending on the time unit of the death series, which could be weekly, monthly, or quarterly. These are some of the sources of variation that could explain the differences between the estimates of excess mortality during the COVID‐19 pandemic that have been provided by different authors (Bilinski and Emanuel 2020; Islam et al. 2021; Jdanov et al. 2021; Karlinsky and Kobak 2021; Kontis et al. 2020; Rizzi and Vaupel 2021; Vestergaard et al. 2020; The Economist DataTeam 2021; Wu and McCann 2020).

Inconsistencies in excess mortality estimates can lead to poor policy decisions and affect the country rankings for excess mortality. Country comparisons are essential for assessing the efficiency of country‐specific policy interventions designed to reduce the impact of the COVID‐19 pandemic. However, changes in country rankings can be partially attributable to the sensitivity of each country to the inputs and methods that are used to estimate excess mortality. To date, little is known about variation in excess mortality estimates resulting from differences in the choice of the mortality index, the reference period, the time unit of the death data, and the method for estimating expected mortality. In addition, the question of how different combinations of these sources of variation can result in different excess mortality estimates remains open. More importantly, the magnitude of these differences has yet to be explored.

When seeking to provide excess mortality estimates, researchers should be aware that this metric depends on the expected mortality level, which can differ depending on the choices made. Thus, this study focuses on four sources of excess mortality variation: (1) the mortality index, (2) the method used to estimate the baseline, (3) the number of years included in the reference period, and (4) the time unit of the death data (weekly and monthly). We investigate to what extent excess mortality estimates depend on these sources of variation. Then, we analyze how important these factors are for each specific country and its excess mortality ranking. To do so, we calculate annual excess mortality estimates for 26 countries in 2020 and create 16 different scenarios that combine two mortality indices, four methods, three reference periods, and weekly and monthly death series to estimate the expected mortality level for 2020. Our findings indicate which of these factors, or which combinations of these factors, have a greater impact on excess mortality estimates within countries, and on the relative positions of countries. In addition, we highlight the importance of carefully choosing inputs and methods for estimating expected mortality before analyzing and drawing substantive conclusions concerning excess mortality.

## Sources of Variation in Excess Mortality Estimates

### Mortality Index

There are different ways to summarize mortality in a population, such as death rates, death counts, and life expectancy. For estimating excess mortality, death rates and death counts are the indices that are most commonly used (Bilinski and Emanuel 2020; Faust et al. 2021; Schöley 2021). In this study, we decide to use death rates mainly for two reasons. First, mortality rates show the intensity of deaths in a population. Second, death rates are a common index when the goal is comparing populations, particularly among those with substantial differences in their population size. Among the death rates researchers have used are crude death rates (CDRs) (Aburto et al. 2021; Basellini et al. 2021; Németh, Jdanov, and Shkolnikov 2021; Stokes et al. 2021), age‐specific death rates (ASDRs) (Németh, Jdanov, and Shkolnikov 2021), and age‐standardized death rates (SDRs) (Islam et al. 2021; Krieger, Chen, and Waterman 2020). These indices reflect different levels and trends in mortality that may result in variation in the expected mortality level used to estimate excess mortality.

Differences in population age structures have a major influence on comparisons of CDRs and death counts between countries. CDRs express real‐life mortality and population losses. However, when comparing levels of mortality across populations, it is desirable to reduce the influence of the age composition by calculating the ASDRs or the SDRs. In this study, we use CDRs and SDRs to estimate excess mortality. Being aware of differences between CDRs and SDRs is key to understanding the differences between excess mortality estimates, which can vary depending on whether an index that is affected by population age structures (CDRs) or an index that controls for differences between age compositions (SDRs) is chosen.

### Method Used to Estimate the Baseline

Using the appropriate method to estimate the baseline is crucial for achieving robust excess mortality estimates. The methods that have been used most frequently for estimating excess mortality during the COVID‐19 pandemic are simple averages and regression models (Basellini et al. 2021; Bilinski and Emanuel 2020; Eurostat 2021; Modig, Ahlbom, and Ebeling 2021; Schöley 2021; The Economist DataTeam 2021; Wu and McCann 2020). In this study, we use four different methods to estimate the baseline mortality (or the expected mortality level in the absence of the pandemic) in order to evaluate the sensitivity of excess mortality estimates to the method chosen, as detailed below.

#### Specific‐Average

The Specific‐Average was notably the most commonly used method for estimating excess mortality among the first excess mortality publications (Krieger, Chen, and Waterman 2020; Modig, Ahlbom, and Ebeling 2021; Stang et al. 2020; The Economist DataTeam 2021; Wu and McCann 2020). Statistical Offices have been using simple averages to compute excess mortality as well (Destatis 2021; Eurostat 2021; Office for Health Improvement and Disparities 2021). The Specific‐Average method is simple to understand and can be easily computed as,

(1) $$y_{t,i}=\frac{1}{r}\cdot \sum_{h=t-r}^{t-1}\alpha_{h,i}+\varepsilon_{t,i},i=1,2,\text{…},n;\;h=t-r,\;t-r+1,\text{…},\;t-1$$

where $y_{t,i}$ is the expected death rate for the year *t* and the week or month *i*, *r* denotes length of the reference period in years, *n* is the total number of weeks or months in the year *t*, $\alpha_{h,i}$ are the observed death rates for the week or month *i* for the year *h*, and $\varepsilon_{t,i}$ are the residuals.

#### Specific‐Average with Trend

The Specific‐Average method does not consider mortality trends. Some researchers have already noted the importance of including time trends in their models for estimating excess mortality (Basellini et al. 2021; Németh, Jdanov, and Shkolnikov 2021; Schöley 2021). Thus, the second model we employ includes linear time trends. To take these trends into account in building the baseline, we added to Equation (1) a term that accounts for the annual trend in death rates as,

(2) $$y_{t,i}=\frac{1}{r}\cdot \sum_{h=t-r}^{t-1}\alpha_{h,i}+\beta h+\varepsilon_{t,i},i=1,2,\text{…},n;\;h=t-r,\;t-r+1,\text{…},\;t-1$$

whereβ is the slope of the linear trend.

#### Harmonic with Trend

The third method is a variant of the Serfling model (Serfling 1963), which considers the seasonality of mortality over a year. This method and its variants are commonly used to estimate excess mortality due to influenza (e.g., Simonsen et al. 2005; Thompson et al. 2003). In this study, we employ the following model:

(3) $$\begin{array}{ccc} y_{t,i} & = & \beta h+\delta \sin\left(2\pi \frac{i}{n}\right)+\eta \cos\left(2\pi \frac{i}{n}\right)+\theta \sin\left(2\pi \frac{i}{\left(\frac{n}{2}\right)}\right)+\upsilon \cos\left(2\pi \frac{i}{\left(\frac{n}{2}\right)}\right)+\varepsilon_{t,i},\; \end{array}$$

where β is the slope of the linear trend, δ,η,θ, and υ are the coefficients that determine harmonic seasonal fluctuations, and $\varepsilon_{t,i}$ are the residuals.

#### Specific‐Trend

The fourth method we employ in this study is used less frequently to estimate excess mortality (Németh, Jdanov, and Shkolnikov 2021). It considers the linear time trend of the week or month *i* of the year *t* and can be computed as

(4) $$y_{t,i}=\beta_{i}h+\varepsilon_{t,i},i=1,2,\text{…},n;\;h=t-r,\;t-r+1,\text{…},\;t-1$$

where $\beta_{i}$ is the slope of the linear trend for the week or month *i*, and $\varepsilon_{t,i}$ are the residuals.

### Reference Period

The reference period, defined as the number of previous years included in the baseline, is also a source of variation across excess mortality estimates. The goal of the baseline is to provide a reference level of mortality that reflects as accurately as possible recent mortality patterns that would have occurred in the absence of the pandemic. Many of the studies on excess mortality due to the COVID‐19 pandemic have arbitrarily chosen the reference period of 2015–19 (Bilinski and Emanuel 2020; Krieger, Chen, and Waterman 2020; Schöley 2021). However, we believe that the choice of the number of years included in the baseline should be based on certain considerations. For instance, for countries that experienced steeper declines in mortality in the early years of the last decade than in the final years of the decade, the expected level of mortality for 2020 will be lower than that based on more recent years if 2010–19 is defined as the reference level instead of a more recent period, such as 2017–19. Thus, the choice of the reference period can affect excess mortality estimates.

In addition, depending on the reference period considered in the baseline, some previous epidemic years may be included in the expected mortality level. In 2015, for instance, European countries experienced a severe influenza epidemic, which resulted in higher levels of mortality in 2015 than in the previous winter season (Fedeli et al. 2017; Nielsen et al. 2019). Thus, if 2015 is included in the reference period, the elevated mortality in that year will increase the mortality level of the baseline. This example highlights the importance of carefully choosing the years included in the reference period used to estimate excess mortality.

To evaluate the sensitivity of excess mortality estimates to changes in the reference period, we consider three different periods: (1) 2010–19, (2) 2015–19, and (3) 2017–19. We have chosen these three reference periods because they reflect, respectively, the mortality level based on a long time series (2010–19), the mortality level based on a more recent mortality pattern (2015–19), and the mortality level in the absence of the acute influenza epidemic of 2015 (2017–19).

### Data Time Unit (Weekly and Monthly Death Series)

Weeks and months are the two time units of the death data that are used most frequently to estimate excess mortality (Jdanov et al. 2021; Karlinsky and Kobak 2021). The use of weekly data provides more precise mortality estimates and the opportunity to obtain information about recent mortality shocks with a minimal delay. However, for several countries, weekly data are not easily available, and monthly data have been used instead. Among the potential pitfalls of using monthly data is that in addition to being a slower way to monitor changes in mortality during a mortality crisis, it provides a smoother time series than using weekly data would. However, little is known about the comparability of excess mortality estimates derived from weekly and monthly data. In this study, we employ weekly and monthly death series to estimate excess mortality, and, in turn, to investigate how the choice of the data time unit affects excess mortality estimates.

### Sensitivity Analysis

Having defined the sources of variation in excess mortality estimates, we evaluate to what extent excess mortality estimates depend on the combination of (1) the mortality index used and (2) the method employed to estimate the baseline. We hypothesized that different combinations of a specific mortality index with a given method could result in different excess mortality estimates. Therefore, we have created eight scenarios in which we apply the four methods to each mortality index. To investigate the sensitivity of the mortality index and the method, we assume, in these eight scenarios, that the reference period is 2015–19, and the data time unit is weekly, as shown in Table 1.

**TABLE 1:** Scenarios for the sensitivity analysis by varying the mortality index and the method

Scenario	Mortality index	Method	Reference period	Time unit
Scenario 1	SDR	Specific‐Average	2015‐2019	Weeks
Scenario 2	SDR	Specific‐Average with Trend	2015‐2019	Weeks
Scenario 3	SDR	Harmonic with Trend	2015‐2019	Weeks
Scenario 4	SDR	Specific‐Trend	2015‐2019	Weeks
Scenario 5	CDR	Specific‐Average	2015‐2019	Weeks
Scenario 6	CDR	Specific‐Average with Trend	2015‐2019	Weeks
Scenario 7	CDR	Harmonic with Trend	2015‐2019	Weeks
Scenario 8	CDR	Specific‐Trend	2015‐2019	Weeks

Source: Authors’ elaboration.

Then, to investigate the impact of the reference period on excess mortality estimates, we have created four additional scenarios, as shown in Table 2. In Scenarios 9–12, we vary the reference period by using 2010–19 and 2017–19 instead of using 2015–19, as in Table 1. We keep constant the method and the time unit of the death series. Scenarios 9–12 are built for both SDRs and CDRs.

**TABLE 2:** Scenarios for the sensitivity analysis by varying reference period

Scenario	Mortality index	Method	Reference period	Time unit
Scenario 9	SDR	Specific‐Average with Trend	2010–2019	Weeks
Scenario 10	SDR	Specific‐Average with Trend	2017–2019	Weeks
Scenario 11	CDR	Specific‐Average with Trend	2010–2019	Weeks
Scenario 12	CDR	Specific‐Average with Trend	2017–2019	Weeks

Source: Authors’ elaboration.

To evaluate the magnitude of the variation in excess mortality by varying the reference period from 2015–19 to 2010–19 and 2017–19, the scenarios presented in Table 2 should be compared with Scenario 2 and Scenario 6 from Table 1. Scenario 2 for SDRs and Scenario 6 for CDRs employ the same method and time unit of Scenarios 9–12, but for the 2015–19 reference period.

Table 3 shows four additional scenarios designed to evaluate the impact on excess mortality of changes in the data time unit. In Scenarios 13–16, we vary the time unit of the death series from weekly to monthly. Unlike in the previous scenarios, we consider weeks 1–52 in leap week years in order to compare monthly with weekly death series, and to avoid potential disagreements between these two time units derived from the 53rd week in leap week years.

**TABLE 3:** Scenarios for the sensitivity analysis by the data time unit

Scenario	Mortality index	Method	Reference period	Time unit
Scenario 13	SDR	Harmonic with Trend	2015–2019	Weeks (1–52)
Scenario 14	SDR	Harmonic with Trend	2015–2019	Months
Scenario 15	CDR	Harmonic with Trend	2015–2019	Weeks (1–52)
Scenario 16	CDR	Harmonic with Trend	2015–2019	Months

Source: Authors’ elaboration.

In Table 3, we combine monthly and weekly (weeks 1–52) data with the Harmonic with Trend method. We have chosen to employ this method because the use of monthly or weekly data combined with the Specific‐Average or Specific‐Average with Trend methods should, theoretically, provide equal excess mortality estimates, given that these methods are mathematically equivalent with respect to the time unit (Karlinsky and Kobak 2021). However, since this is not the case for the Harmonic with Trend method, we believe that differences in excess mortality may emerge when this method is combined with different time units.

To investigate the magnitude of variations in excess mortality estimates by using monthly instead of weekly data, we compare the scenarios in Table 3 within each mortality index. For instance, the magnitude of the variation in excess mortality by using monthly instead of weekly data for the SDRs, the Harmonic with Trend method, and the 2015–19 reference period, is equal to the difference between the excess mortality levels estimated by Scenario 13 and Scenario 14.

All excess mortality rates come along with 95 percent confidence intervals derived from Monte Carlo simulations based on the assumption that deaths are binomially distributed with population counts being an offset. We performed 1,000 iterations for each country–year–week/month‐specific estimates. This method is detailed in Andreev and Shkolnikov (2010).

### Data

We used data from the Short‐Term Mortality Fluctuations (STMF) data series (Jdanov et al. 2021), which is a new component of the Human Mortality Database (HMD) (Barbieri et al. 2015). We draw data from 26 countries/regions. Of these 26 countries/regions, 23 have complete series lasting from 2010 to 2020, including Austria, Belgium, Denmark, England and Wales, Estonia, Finland, France, Hungary, Israel, Latvia, Lithuania, the Netherlands, Norway, Poland, Portugal, Republic of Korea, Scotland, Slovenia, Slovakia, Spain, Sweden, Switzerland, and Taiwan. In Italy, New Zealand, and the United States, the time series start from 2011, 2011, and 2015, respectively.

Weekly deaths are directly drawn from the STMF data series, which follow the ISO 8601‐2004 guidelines (Jdanov et al. 2021). Monthly deaths are collected from the national statistical offices. The figures are published by these offices, or they are requested by the STMF team. Monthly deaths are available for the 26 countries. Appendix A in the Supplemental Materials presents the list of monthly data sources by country.

Population exposures by ISO 8601‐2004 (seven‐day weeks) are retrieved from the STMF data series. The STMF calculates weekly population exposures by dividing annual population exposures by the number of weeks in a year under the assumption of zero migration (Jdanov et al. 2021). We followed this approach to calculate monthly population exposures as the annual exposures divided by 12.

From the weekly and monthly data, we calculate annual mortality rates for all ages and men and women together. The HMD core provides annual death rates from 2010 to 2018 for all the countries considered in this study, except Hungary (2010–17), Israel (2010–16), New Zealand (2010–13), Slovenia (2010–17), and Slovakia (2010–17). The available HMD annual death rates were forecasted up to 2020 (Jdanov et al. 2021). However, since the STMF data series are preliminary, some deaths may not be included in the weekly series. In addition, because the STMF data series use ISO weeks (each week has seven days), the death rates from the STMF might be slightly different from the annual death rates derived from the HMD core. Therefore, we compare annual death rates from both the STMF data series and the HMD core data, and then adjust for any differences that might emerge, while assuming that the HMD core data represent the gold standard.

To make the annual death rates derived from weekly data comparable across leap week years (years with 53 weeks) and non–leap week years (years with 52 weeks), we calculate baseline mortality levels based on weeks 1–52 and assume that the baseline for week 52 is equal to that of week 53. The monthly death counts were adjusted as well. Months in leap years have, on average, 30.50 days (366/12), while the average number of days per month in non‐leap years is 30.42 (365/12). We assume that the average number of days in both leap and non‐leap years is 30.44 days (365.25/12). Thus, we multiply the number of deaths in each month by the ratio between 30.44 and the actual number of days in each month. Then, to make sure that the total number of deaths in a year did not change, we adjust for any eventual differences in the annual total number of deaths before and after considering that the average number of days in a month is 30.44.

To age‐standardize the CDRs, we use the European Population Standard of 2013 (European Commission 2013) and apply the methodology developed by Klimkin, Shkolnikov, and Jdanov  (2021) that combines the aggregate weekly death series with the detailed population and annual death data.

## Results

**FIGURE 1:**
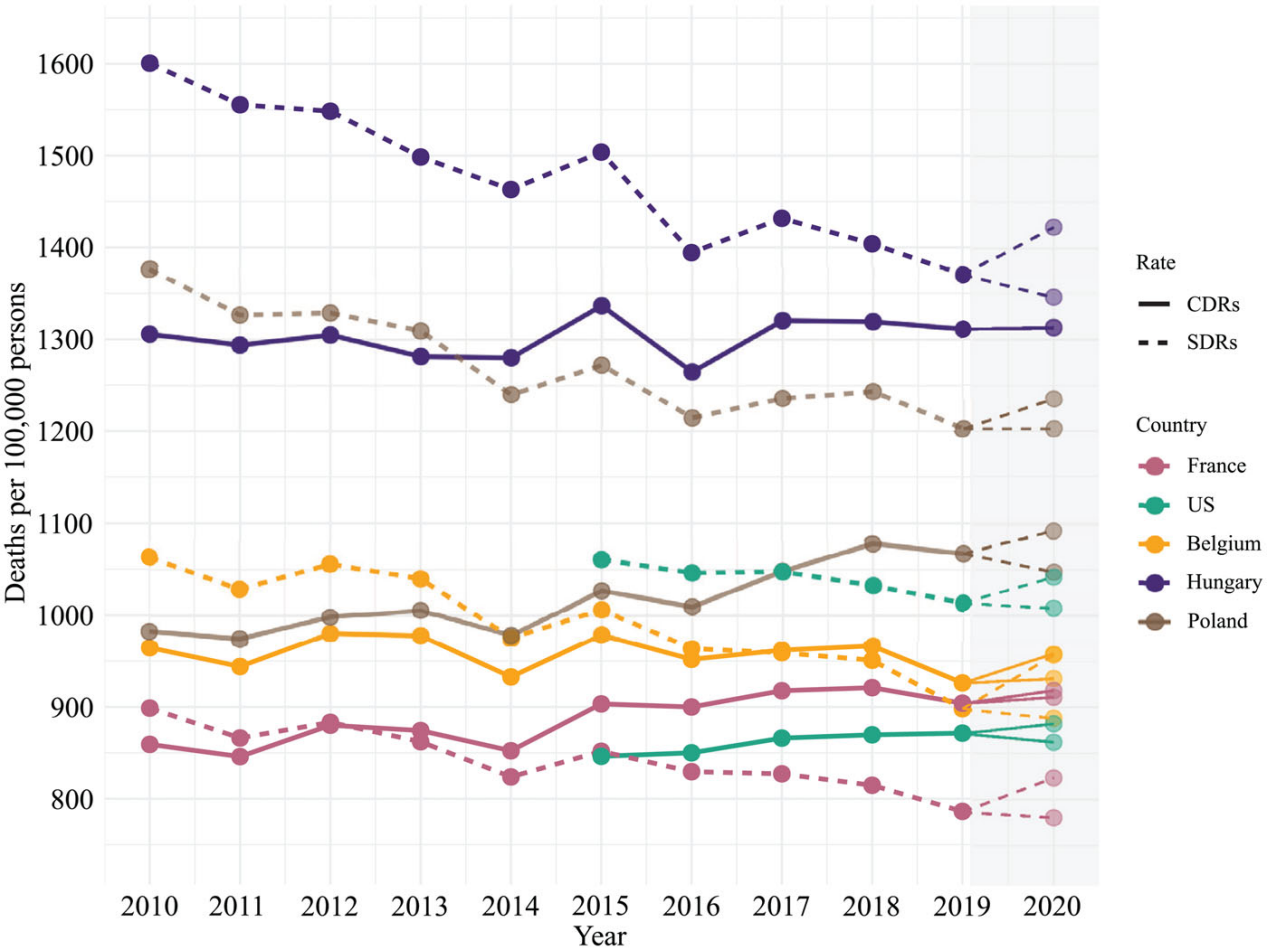
Observed CDRs and SDRs in France, the United States, Belgium, Hungary, and Poland in 2010–2019, and their expected values in 2020 NOTES: The values for 2020 are the expected death rates derived from Scenario 1 and Scenario 5 (Specific‐Average) for the SDRs and Scenario 2 and Scenario 6 (Specific‐Average with Trend) for the CDRs. These scenarios are based on the 2015–2019 reference period and weekly data. The gray area highlights the expected values. SOURCES: Jdanov et al. (2021) and European Commission (2013).

Figure 1 shows the levels and trends in observed death rates between 2010–19 and highlights variations in the estimated levels of expected mortality in 2020 for France, the United States, Belgium, Hungary, and Poland. The figure shows temporal changes in the observed CDR and SDR values up to 2019, and two values of the expected rates depending on the method used to estimate the baseline (Specific‐Average and Specific‐Average with Trend) in 2020.

Figure 1 introduces our later analysis by showing that both the levels and the trends change considerably with the mortality index used between 2010 and 2019. In the United States, Poland, and Hungary, the SDRs are found to be much higher than the CDRs. In 2015, for instance, the SDR was 25 percent higher than the CDR in the United States and was 13 percent higher than the CDR in Hungary. Figure 1 also reveals a striking level of variation in the trends of the two indices. In France and in the United States, the CDR increases over time, while the SDR decreases. In Hungary, the SDR decreases, while the CDR remains nearly stable between 2017 and 2019. The differences in the levels and trends in the CDRs and the SDRs depending on the methods used resulted in variation in the expected level of mortality in 2020, as the gray area of Figure 1 shows.

### Sensitivity of Excess Mortality Estimates to Mortality Index and Method

**FIGURE 2:**
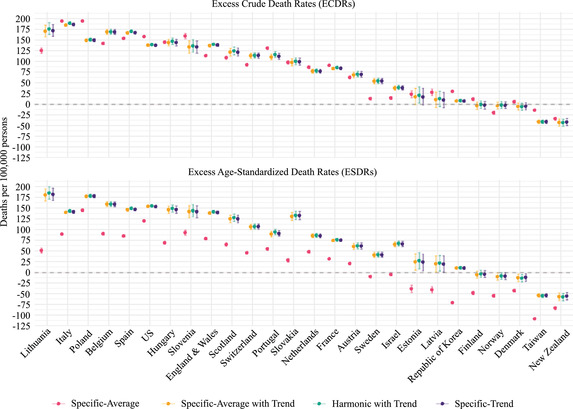
Excess mortality rates by varying the method for each mortality index and country, 2020 NOTES: The reference period is 2015–2019, and the data time unit is weekly (see Table 1 for more details). 95% confidence intervals are based on Monte Carlo simulation. SOURCES: Jdanov et al. (2021) and European Commission (2013).

Figure 2 illustrates in greater detail the impact on excess mortality of the baseline variations presented in Figure 1. Figure 2 displays the excess mortality rates by varying the method used to calculate the baseline for both the CDR and the SDR. The figure demonstrates that different combinations of the mortality index and the method used result in different excess mortality estimates for all countries, as was already shown in Figure 1. Figure 2 illustrates the impact of the method used on the crude death rate of excess mortality (ECDRs) and the age‐standardized death rate of excess mortality (ESDRs). For both mortality indices, the three methods that consider time trends (Specific‐Average with Trend, Harmonics with Trend, and Specific‐Trend) estimate similar excess mortality rates for all countries, while the Specific‐Average is the method that provides excess mortality estimates that tend to result in greater variation. For the CDRs, the Specific‐Average method can produce higher or lower levels of excess mortality than the other methods. In Italy, Poland, and the United States, the Specific‐Average method provides higher excess mortality rates than the other methods; while in Sweden, Israel, and Norway, the Specific‐Average method provides lower excess mortality rates than the other methods. For the SDRs, the Specific‐Average method systematically provides lower excess mortality rates for all countries than the methods that account for linear trends. In Lithuania, for instance, the excess mortality for SDR estimated by the Specific‐Average method is nearly four times lower than that derived from the other methods. Table 1B in Appendix B (Supplemental Materials) shows the excess mortality values presented in Figure 2.

**FIGURE 3:**
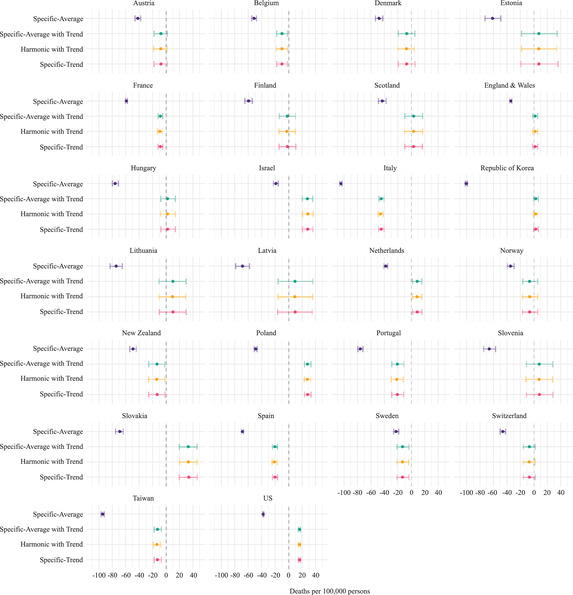
Differences between the excess age‐standardized death rates (ESDRs) and the excess CDRs (ECDRs) values for each method used to estimate the baseline mortality, 2020 NOTES: The differences are computed as excess SDR minus excess CDR. The reference period is 2015–2019, and the data time unit is weekly (see Table 1 for more details). 95% confidence intervals are based on Monte Carlo simulation. SOURCES: Jdanov et al. (2021) and European Commission (2013).

To isolate the effect of the mortality index on excess mortality estimates and to highlight the magnitude of its variation, Figure 3 presents the difference between the excess mortality rates estimated by using the SDRs and the CDRs across the four methods. Figure 3 shows substantial differences between excess mortality rates when the mortality index is varied within each method for all countries. The greatest differences emerge when the Specific‐Average method is employed for all countries, except for Israel. In Italy and the Republic of Korea, the magnitude (or absolute values) of variations in excess mortality rates (per 100,000 persons) due to the use of the CDRs or the SDRs combined with the Specific‐Average method are above 100 deaths per 100,000 persons. In Israel, the Specific‐Average is the method that results in the lowest absolute difference in excess mortality rate when the mortality index varies. For all countries, the magnitude of the differences in the excess mortality rates depending on the mortality index used is smaller for the methods that account for trends. For example, in Hungary, Finland, and Scotland, there is virtually no variation in the excess mortality rates depending on the use of the CDRs or the SDRs for all baseline mortality levels that account for the trend.

### Sensitivity of Excess Mortality Estimates to the Reference Period and the Data Time Unit

**FIGURE 4:**
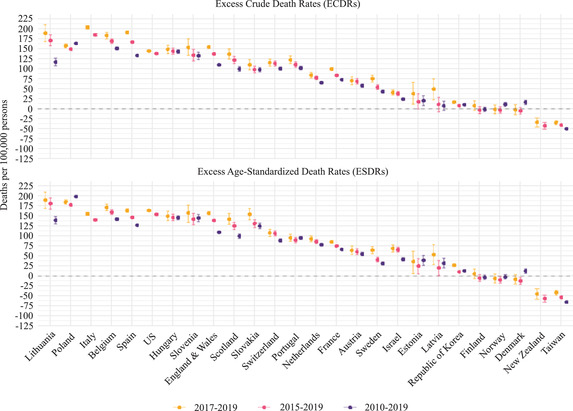
Excess mortality rates by varying the reference period for each mortality index and country, 2020 NOTES: The method used to estimate the baseline is the Specific‐Average with Trend, and the data time unit is weekly. The Italian and the New Zealand data series start in 2011, and the US data series start in 2015. 95% confidence intervals are based on Monte Carlo simulation. SOURCES: Jdanov et al. (2021) and European Commission (2013).

Figure 4 shows that excess mortality rates can vary by changing the reference period for each mortality index using the Specific‐Average with Trend method (Scenarios 9–12). This figure suggests that when the reference period is longer, the excess mortality rates are lower for most countries. However, the excess mortality estimates depend on the trend of the mortality index in the chosen reference period. In Belgium, for instance, where there was a steeper decline in the SDRs between 2015–19 than in the 2010–19 reference period (Figure 1), the excess mortality rate derived from the 2010–19 reference period was 20 percent lower than that derived from the 2017–19 reference period. Poland experienced a different SDR trend than Belgium (Figure 1). In Poland, the decline in the SDRs was steeper between 2010 and 2019 than it was between 2015 and 2019 (Figure 1). As a result of this trend, excess mortality rates appear to be higher in Poland when the 2010–19 period is considered rather than the 2015–19 period. Table 2B in Appendix B presents all of the excess mortality values displayed in Figure 4.

**FIGURE 5:**
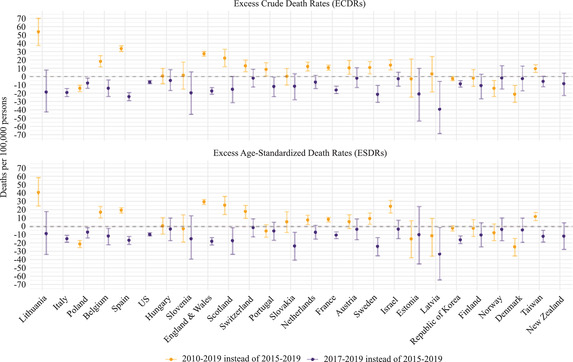
Differences in excess mortality rates by varying the reference period for each mortality index and country, 2020 NOTES: The differences are computed as excess mortality derived from the 2015–2019 reference period minus excess mortality derived from 2010–2019 or 2017–2019 reference period. The method used to estimate the baseline is the Specific‐Average with Trend, and the data time unit is weekly. The Italian and the New Zealand data series starts in 2011, and the US data series starts in 2015. 95% confidence intervals are based on Monte Carlo simulation. SOURCES: Jdanov et al. (2021) and European Commission (2013).

Figure 5 shows the differences in the estimated excess mortality when the reference period for each death rate is varied. The magnitude (or the absolute value) of the differences in excess mortality rates changes substantially within countries when the reference period is changed, with the point estimate ranging from 0.1 to 55 deaths (per 100,000 persons). Moreover, the magnitude of the differences within countries varies when the mortality index is combined with the reference period. By changing the reference period from 2010–19 to 2015–19, the magnitude of the point estimate difference between excess mortality rates in Lithuania is 54 deaths (per 100,000 persons) for the ECDR and 40 deaths (per 100,000 persons) for the ESDR. For Portugal, Figure 5 presents a different pattern: for the ESDR, the differences are very small depending on whether the 2010–19 or the 2017–19 reference period is used instead of the 2015–19 reference period; while for the ECDR, the magnitude of the variation in the excess mortality estimates is slightly higher when the 2017–19 reference period is used instead of the 2015–19 reference period.

**FIGURE 6:**
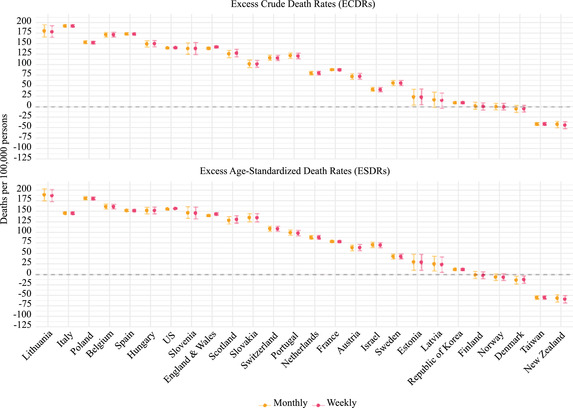
Excess mortality rates by varying the time unit of the death series for each mortality index and country, 2020 NOTES: The method used to estimate the baseline is the Harmonic with Trend, and the reference period is 2015–2019. 95% confidence intervals are based on Monte Carlo simulation. SOURCES: Jdanov et al. (2021) and European Commission (2013).

Figure 6 compares excess mortality rates depending on whether weekly or monthly death series are used in combination with the Harmonic with Trend method (scenarios 13–16). The excess mortality rates are very similar for both data time units across all countries and for both death rates.

**FIGURE 7:**
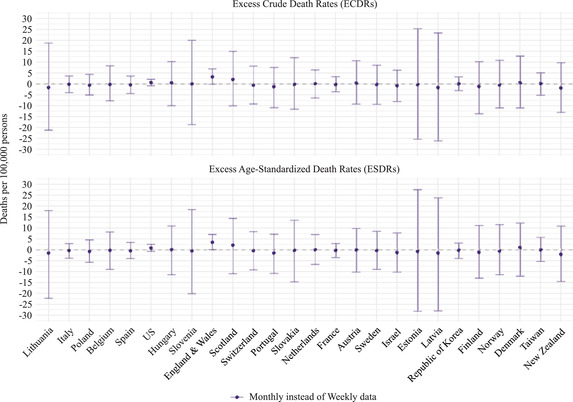
Differences in excess mortality rates by using monthly instead of weekly data, for each mortality index and country, 2020 NOTES: The differences are computed as excess mortality derived from the weekly death series minus excess mortality derived from the monthly death series. The method used to estimate the baseline is the Harmonic with Trend, and the reference period is 2015–2019. 95% confidence intervals are based on Monte Carlo simulation. SOURCES: Jdanov et al. (2021) and European Commission (2013).

Figure 7 complements Figure 6 by showing the excess mortality differences by using monthly instead of weekly data. The magnitude (or absolute value) of the point estimate for the difference is very small across all countries and for both ESDRs and ECDRs (below 3.5 deaths per 100,000 persons). In France, for instance, the change in excess mortality rates is below 0.5 deaths (per 100,000 persons). In countries like Lithuania, Slovenia, Estonia, and Latvia, the point estimate for the difference is also very small, but confidence intervals are wide. In Estonia, for instance, the point estimate for the difference is lower than 1 death (per 100,000 persons), but the upper and the lower bounds can be above 25 deaths (per 100,000 persons). The largest magnitude of the point estimate for the difference is in England and Wales, where the variation in the excess mortality rate is about 3.5 deaths (per 100,000 persons).

As we mentioned in the Sensitivity Analysis section, theoretically, excess mortality estimated with the Specific‐Average with Trend method should not vary when the data time unit is changed. To empirically test this theoretical claim, we compare the results shown in Figure 7 with excess mortality rates when the Specific‐Average with Trend method is employed instead of the Harmonic with Trend method. Figure 1C in Appendix C (Supplemental Materials) presents this comparison. As expected, Figure 1C shows that the magnitude of the point estimate for the differences in excess mortality rates is generally lower when the Specific‐Average Trend method is used. However, in this comparison, we use a reference period that includes the 2015 leap year, which can lead to discrepancies between monthly and weekly data. Thus, Figure 1C also presents the variation in excess mortality due to the data time unit when the Specific‐Average Trend method is combined with the 2017–19 reference period. This latter combination results in virtually no variation in the point estimate for the differences between excess mortality rates depending on whether monthly or weekly data are used. Thus, Figure 1C suggests that both the method employed and the use of leap years in the reference period lead to variations in excess mortality derived from monthly or weekly death series.

### Country ranking

**FIGURE 8:**
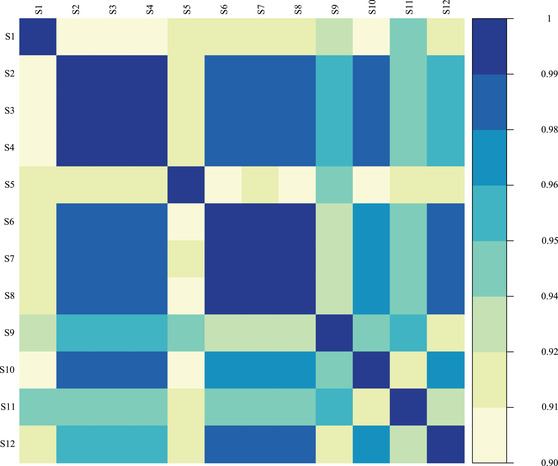
Spearman's correlation coefficients between excess mortality rankings for the 12 scenarios across the 26 countries, 2020 SOURCE: Authors’ elaboration.

Figure 8 presents Spearman's correlation coefficients between excess mortality rankings for Scenarios 1–12 (S1–S12) across the 26 countries. Scenarios 13–16, which evaluate variations in excess mortality rates resulting from the choice of the data time unit, were not included in this analysis because they do not consider the 53rd week of leap week years in the baseline, while other scenarios account for that week. This figure highlights the similarities and differences in the countries’ excess mortality rankings estimated for each scenario presented in Tables 1, 2. Darker blue hues indicate a stronger correlation between the excess mortality pair comparisons.

The Spearman's correlation coefficients for different pairs of excess mortality rates are above 0.9, which indicates a high degree of correlation between the excess mortality estimates (Figure 8). A lower degree of correlation is observed in pair comparisons that involve Scenario 1 (S1) and Scenario 5 (S5). Scenario 1 for the ESDR and Scenario 5 for the ECDR employ the only method that does not account for the trend, the Specific‐Average method (see Table 1 for more details). This finding highlights the impact of choosing a method with or without a linear trend on country rankings. Discrepancies in rankings are also observed when scenarios that consider the 2010–19 reference period (S9 and S11) are combined with the other scenarios. On the other hand, the highest correlation in rankings is observed in scenarios in which the method accounts for the trend term. This finding suggests that for a given mortality index and reference period, employing different methods that consider linear trends provides similar country rankings.

In addition to the Spearman's correlation coefficients shown in Figure 8, Table 4B in Appendix B presents the country rankings for excess mortality rates from the highest to the lowest for each country and across all scenarios. This table shows some country‐specific changes in the country rankings. For instance, when moving from Scenario 1 to Scenario 2, which consider the Specific‐Average method and the Specific‐Average with Trend method, respectively, Lithuania rises from the eleventh to the first position, while Slovakia rises from the fifteenth to the tenth. The magnitude of the difference between excess mortality rates derived from Scenario 1 and Scenario 2 is 129.5 and 102.3 deaths (per 100,000 persons), respectively, for Lithuania and Slovakia. For Italy, Table 4B shows a marked change in the ranking when CDR (Scenarios 6–8) is employed instead of SDR (Scenarios 2–4): i.e., Italy rises to the first position when CDR is used, and the magnitude of the difference in the excess mortality rate when CDR is used instead of SDR is about 45 deaths (per 100,000 persons).

## Discussion

We investigated the sensitivity of excess mortality estimates in 2020 to the choice of the mortality index, the method, the reference period, and the time unit of the death series in 26 countries/regions. Our results showed that excess mortality rates varied substantially when these factors changed, and the magnitude of these variations changed markedly within countries, which resulted in changes in the country rankings.

The choice of the mortality index was found to be one of the main sources of excess mortality variation within countries. We used two mortality indices that provided excess mortality estimates with and without the influence of the population age structure. We showed that both the levels and the trends differed substantially for the CDRs and the SDRs, which provided diverse baseline mortality levels, and which, in turn, led to variations in the excess mortality estimates.

In the context of population aging, whether the CDR or the SDR is used is crucial for country comparisons. Differences between population age structures are confounding factors when the goal is to compare the populations' mortality levels (Preston, Heuveline, and Guillot 2001). A higher proportion of older adults combined with the steep age gradient in COVID‐19 mortality (Goldstein and Lee 2020) (considering that excess mortality was largely driven by deaths due to SARS‐CoV‐2) resulted in higher CDRs for older than for younger populations. Italy is a good example of this phenomenon, because it has one of the oldest populations in the world (Gesano and Strozza 2011; Mazzola et al. 2016; Murphy 2017). Our findings showed that the magnitude of the variation in the excess mortality rate changed markedly, depending on whether the CDR or the SDR was used. As a result, there were striking variations in the Italian position in country rankings, with Italy rising to the top ranking when the CDR was used.

The method used to estimate the baseline also appears to be an important source of variation in excess mortality. We showed that methods that considered linear trends provided similar excess mortality rates for both the CDR and the SDR. However, we also found that the Specific‐Average method produced the lowest excess mortality rates across all countries for the SDR, which is in line with the findings of Schöley (2021). However, while a similar pattern was observed across all countries, the magnitude of the variation in excess mortality rates due to the choice of a method that did or did not account for trends changed for each country, ranging from about 130 deaths (per 100,000 persons) in Lithuania to 26 deaths (per 100,000 persons) in New Zealand. On the other hand, we found no similar patterns across countries when the CDR was combined with the Specific‐Average method. In addition, our findings provided further evidence that the choice of method matters in order to understand variations in excess mortality, as we found that the magnitude of these variations was country‐specific and depended on the selection of the mortality index.

It is also relevant to highlight that we employed mainstream methods to calculate the baseline mortality level to date. Our goal was not to provide the best model; a cross‐validation analysis is needed to do that. Instead, our objective was to assess the magnitude of the variation in excess mortality due to the choice of one of the most mainstream methods used to calculate baseline mortality. The final choice of the model should be task‐specific and take into account this variation.

In addition, we observed that the choice of the reference period also matters when estimating excess mortality. In contrast with previous research on excess mortality in which the reference period was arbitrarily chosen (Bilinski and Emanuel 2020; Karlinsky and Kobak 2021; Schöley 2021), we showed that there were important variations in excess mortality estimates depending on the reference period selected. Our findings indicated that for most countries, a longer reference period resulted in lower excess mortality. More importantly, we highlighted the relevance of the trend of the mortality index in the chosen reference period, especially when methods for estimating the baseline accounted for the trends.

Furthermore, our analysis found that the data time unit of the death series was the factor associated with the smallest variations in annual excess mortality estimates. This finding is relevant since some countries released only monthly data; thus, some sources of excess mortality mixed monthly with weekly death series in their country comparisons (Karlinsky and Kobak 2021; The Economist DataTeam 2021). Using different time units might lead to incorrect results for a short period (e.g., month) because of the shift in the reference period. For example, the period that refers to the first four weeks might start between 29 December and 3 January while the first month (January) always has fixed dates. Thus, the time frame used to estimate expected mortality will differ.

We showed all the steps needed to make weekly and monthly death series comparable to achieve similar excess mortality estimates. The magnitude of the impact of the data unit on excess mortality estimates depended on both the method used and the presence of leap week years in the reference period. The choice of a method that is equivalent regarding the time unit, such as the Specific‐Average or the Specific‐Average with Trend, reduced the impact of the data unit chosen on variations in excess mortality. Indeed, when the Specific‐Average with Trend method was combined with a reference period that did not include leap week years in the baseline, there was virtually no changes in excess mortality depending on whether monthly or weekly data were used. Nonetheless, caution is still needed when excess mortality levels estimated from different data time units are compared, especially if one employs the Harmonic with Trend method combined with the 2015–19 reference period.

Based on our findings, we provide guidelines for estimating excess mortality. The first recommendation is related to the mortality index. When comparing populations, differences in their age compositions should be considered; thus, age‐standardized rates are recommended. However, it is also important to highlight that age‐standardized rates are hypothetical rates that express what would have been observed in a population if it had the same age composition as the standard population. By contrast, CDRs express real‐life mortality and population losses; as such, they are not misleading when populations are not compared. The last note is also valid for comparison within the national population. For example, using CDRs to estimate sex differences in COVID‐related losses leads to significant underestimation. Thus, we recommend using age‐standardized indicators as an additional measure in all cases when crude rates are used. Our second recommendation regards the methods for estimating the baseline mortality. These methods should consider mortality trends over time. All of the methods we employed that considered linear trends estimated similar excess mortality rates, while the Specific‐Average method that resulted in the greatest variation and tended to underestimate population losses. The third suggestion is for the reference period. The number of years included in the baseline period should be large enough to identify a stable and clear mortality trend. Therefore, the best length for the reference period is country‐ and model‐specific. Our last recommendation is for the comparability of different time units of the death series. To compare weekly and monthly death series, some data adjustments are needed. Moreover, the method used and the reference period (which may or may not include leap week years) should both be chosen carefully. Finally, there is a trade‐off between the quality of estimates and their comparability across time and space. Countries have been experiencing different changes in mortality over time, which should be taken into account when estimating excess mortality.

This study is just the first step in revealing how sensitive excess mortality estimates are as a result of specific choices. Here, we showed variations in excess mortality estimates at the aggregated level. However, further analysis disaggregated by sex and age is needed as well, and may reveal a stronger variation for some specific ages and sex. We hope our analysis will shed some light on the complexity of estimating excess mortality. Moreover, if the COVID‐19 pandemic continues or a new pandemic/epidemic emerges, our study will help researchers to better measure the full mortality impact of the mortality crisis and consequently provide more efficient guidelines to health policymakers.

## Supporting information


**Table 1B**: Excess mortality rates (per 100,000 persons) for scenarios 1–8 by country, 2020.
**Table 2B**: Excess mortality rates (per 100,000 persons) for scenarios 2, 6, 9–12 by country, 2020
**Table 3B**: Excess mortality rates (per 100,000 persons) for scenarios 13–16 by country, 2020.
**Table 4B**: Excess mortality rates (per 100,000 persons) and country ranking (in parentheses) for each scenario, 2020.
**Figure 1C**: Differences between excess mortality rates by using monthly instead of weekly data for each mortality index and country, 2020
