# Supplementary material for: Sensitivity Analysis of Excess Mortality due to the COVID‐19 Pandemic
Source: Popul Dev Rev. 2022 Mar 3;48(2):279–302. doi: 10.1111/padr.12475 (PMC9115405; doi:10.1111/padr.12475)
Supplement: Supplementary file 1 — Table 1B: Excess mortality rates (per 100,000 persons) for scenarios 1–8 by country, 2020. Table 2B: Excess mortality rates (per 100,000 persons) for scenarios 2, 6, 9–12 by country, 2020 Table 3B: Excess mortality rates (per 100,000 persons) for scenarios 13–16 by country, 2020. Table 4B: Excess mortality rates (per 100,000 persons) and country ranking (in parentheses) for each scenario, 2020. Figure 1C: Differences between excess mortality rates by using monthly instead of weekly data for each mortality index and country, 2020 [file PADR-48-279-s001.docx]

**Supplemental Materials**

**Appendix A**

*List of monthly data sources by country*

**Austria**. Statistik Austria: Deceased by demographic criteria. Available at <https://statcube.at/statistik.at/ext/statcube/jsf/tableView/tableView.xhtml> (accessed on 06.03.2021)

**Belgium**. STATBEL: Number of deaths per day, sex, age, region, province, district 2009-2021. Available at <https://statbel.fgov.be/en/open-data/number-deaths-day-sex-district-age> (accessed on 03.03.2021)

**Denmark**. Statistics Denmark: Deaths by day of death and month of death (2007-2020). Available at <https://www.statbank.dk/statbank5a/SelectVarVal/Define.asp?MainTable=DODDAG&PLanguage=1&PXSId=0&wsid=cftree> (accessed on 03.03.2021)

**England & Wales**. Office for National Statistics: Monthly figures on deaths registered by area of usual residence, 2006-2020. Available at <https://www.ons.gov.uk/peoplepopulationandcommunity/birthsdeathsandmarriages/deaths/datasets/monthlyfiguresondeathsregisteredbyareaofusualresidence> (accessed on 03.03.2021)

**Estonia.** Statistics Estonia: Dataset:RV04: Preliminary data of registration of deaths by month and county of the registration. Available at <https://andmed.stat.ee/en/stat/rahvastik__rahvastikusundmused__surmad/RV04> (accessed on 04.03.2021)

**Finland**. Statistics Finland: 12ah -- Deaths by month, 1945-2020. Available at <https://pxnet2.stat.fi/PXWeb/pxweb/en/StatFin/StatFin__vrm__kuol/statfin_kuol_pxt_12ah.px/> (accessed on 06.03.2021)

**France**. INSEE, L’Institut national de la statistique et des études économiques: Demography - Number of deaths - Metropolitan France, 1946-2021. Available at <https://www.insee.fr/en/statistiques/serie/000436394> (accessed on 23.02.2021)

**Hungary**. Hungarian Central Statistical Office: Main indicator of vital events (monthly data, 2017-2021). Available at <http://www.ksh.hu/stadat_files/nep/en/nep0064.html> (accessed on 06.03.2021)

Hungarian Central Statistical Office: Deaths in reference year (1995-2019). Available at <http://statinfo.ksh.hu/Statinfo/haDetails.jsp?query=kshquery&lang=en> (accessed on 06.03.2021)

**Israel**. Central Bureau of Statistics. Deaths of Israeli Residents, by Year and Month, 2000-2021 - All Ages. Data obtained by the CBS Series Generator on the topic of Population, Subtopic of Marriages, Divorces, Live-Births and Deaths. Available at <https://boardsgenerator.cbs.gov.il/pages/Sdarot/wizardpage.aspx?level_1=2&level_2=2&level_3=1&level_4=1&level_5=644&l=1> (accessed on 04.03.2021)

**Italy**. ISTAT: Dataset con i decessi giornalieri in ogni singolo comune di residenza. Available at <https://www.istat.it/it/archivio/240401> (accessed on 05.03.2021)

**Latvia**. Central Statistical Bureau of Latvia. IE040m. Live births and deaths by sex and by month. Available at <https://data.stat.gov.lv/pxweb/en/OSP_PUB/START__POP__IM__IMSV/IDS010m> (accessed on 03.03.2021)

**Lithuania**. Statistics Lithuania: Monthly and Weekly Demographic Indicators: Deaths by month. Available at <https://osp.stat.gov.lt/statistiniu-rodikliu-analize#/> (accessed on 25.02.2021)

**The Netherlands**. Statistics Netherlands (CBS): Population dynamics (Deaths) by month and year. Available at <https://opendata.cbs.nl/statline/#/CBS/en/dataset/83474ENG/table?ts=1623842358795> (accessed on 24.02.2021)

**New Zealand**. Stats NZ: Monthly death registrations by ethnicity-age-sex: January 2010 to December 2020. Available at <https://www.stats.govt.nz/information-releases/births-and-deaths-year-ended-december-2020-including-abridged-period-life-table> (accessed on 25.02.2021)

**Norway**. Statistics Norway: Deaths. Preliminary figures, by sex, year, month, age, contents and region. Available at <https://www.ssb.no/en/statbank/table/12982> (accessed on 03.03.2021)

**Poland**. Statistics Poland: Statistical Bulletin No 12/2020,Tabl.7. Population and vital statistics. Available at <https://stat.gov.pl/en/topics/other-studies/informations-on-socio-economic-situation/statistical-bulletin-no-122020,4,120.html> (accessed on 03.03.2021)

**Portugal**. Statistics Portugal: Deaths by place of residence and sex, monthly. Available at <https://www.ine.pt/xportal/xmain?xpid=INE&xpgid=ine_indicadores&indOcorrCod=0007264&contexto=bd&selTab=tab2> (accessed on 03.03.2021)

**Republic of Korea**. KOSIS: Vital statistics (deaths) by Month for Provinces. Available at <https://kosis.kr/statHtml/statHtml.do?orgId=101&tblId=DT_1B8000G&language=en&conn_path=I3> (accessed on 25.02.2021)

**Scotland**. National Records of Scotland. Deaths in Scotland by month of registration and NHS Board area, 1990 – 2021. Available at <https://www.nrscotland.gov.uk/statistics-and-data/statistics/statistics-by-theme/vital-events/general-publications/weekly-and-monthly-data-on-births-and-deaths/monthly-data-on-births-and-deaths-registered-in-scotland> (accessed on 03.03.2021)

**Slovenia**. Republic of Slovenia Statistical Office, SIStat: Deaths by day of death, Slovenia, monthly. Available at <https://pxweb.stat.si/SiStatData/pxweb/en/Data/-/05L1018S.px/> (accessed on 25.02.2021)

**Slovakia**. Statistical Office of the Slovak Republic. Deaths by Month of Death, Age, Sex and Causes of Death - SR-Area-Reg (monthly). Available at the DataCube <http://datacube.statistics.sk/#!/view/en/VBD_SK_WIN2/om3801mr/v_om3801mr_00_00_00_en> (accessed on 04.03.2021).

**Spain**. Instituto Nacional de Estadística: Defunciones por edad, mes y sexo. Definitivos (2010-2019) and Provisionales (2020). Available at <https://www.ine.es/dyngs/INEbase/es/operacion.htm?c=Estadistica_C&cid=1254736177008&menu=resultados&idp=1254735573002> (accessed on 08.03.2021)

**Sweden**. Statistics Sweden, SCB: Deaths per month by region, Region of birth, age and sex. 2000-2020. Available at <https://www.statistikdatabasen.scb.se/pxweb/en/ssd/START__BE__BE0101__BE0101I/DodaManadReg/> (accessed on 08.03.2021)

**Switzerland**. Office Fédéral de la Statistique: Décès par mois et mortalité depuis 1803 selon Année et Caractéristique démographique et indicateur. Available at <https://www.bfs.admin.ch/asset/fr/px-x-0102020206_111> (23.02.2021)

**Taiwan.** Dept. of Household Registration Affairs, MOI. Number and Rates of Birth, Death, Marriage and Divorce. Available at <https://www.ris.gov.tw/app/en/3911> (accessed on 06.03.2021)

**U.S.A**. National Center for Health Statistics, CDC-NCHS: Deaths by month tabulated from the Mortality Multiple Cause Files. <https://www.cdc.gov/nchs/data_access/vitalstatsonline.htm#Mortality_Multiple> (accessed on 25.02.2021)

**Appendix B**

*Supplementary Tables*

**Table 1B:** Excess mortality rates (per 100,000 persons) for Scenarios 1-8 by country, 2020.

| **Country** | **Index** | **Method** | **Reference Period** | **Time Unit** | **Excess Mortality** | **Confidence Interval** | |
| --- | --- | --- | --- | --- | --- | --- | --- |
|  |  |  |  |  |  | **Lower limit** | **Upper limit** |
| Austria | CDR | Specific-Average | 2015-2019 | Weekly | 62.85 | 59.94 | 65.75 |
| Austria | CDR | Specific-Average with Trend | 2015-2019 | Weekly | 68.45 | 62.05 | 75.56 |
| Austria | CDR | Harmonic with Trend | 2015-2019 | Weekly | 70.17 | 63.66 | 77.25 |
| Austria | CDR | Specific-Trend | 2015-2019 | Weekly | 69.73 | 63.26 | 76.97 |
| Austria | SDR | Specific-Average | 2015-2019 | Weekly | 20.74 | 17.72 | 23.67 |
| Austria | SDR | Specific-Average with Trend | 2015-2019 | Weekly | 60.6 | 53.5 | 68.03 |
| Austria | SDR | Harmonic with Trend | 2015-2019 | Weekly | 62.18 | 55.1 | 69.32 |
| Austria | SDR | Specific-Trend | 2015-2019 | Weekly | 61.83 | 54.62 | 69.34 |
| Belgium | CDR | Specific-Average | 2015-2019 | Weekly | 142.28 | 139.83 | 144.78 |
| Belgium | CDR | Specific-Average with Trend | 2015-2019 | Weekly | 169.25 | 163.54 | 175.09 |
| Belgium | CDR | Harmonic with Trend | 2015-2019 | Weekly | 169.52 | 163.91 | 175.44 |
| Belgium | CDR | Specific-Trend | 2015-2019 | Weekly | 169.02 | 163.21 | 175.03 |
| Belgium | SDR | Specific-Average | 2015-2019 | Weekly | 90.69 | 88.17 | 93.1 |
| Belgium | SDR | Specific-Average with Trend | 2015-2019 | Weekly | 159.32 | 153.69 | 165.04 |
| Belgium | SDR | Harmonic with Trend | 2015-2019 | Weekly | 159.38 | 153.81 | 165.18 |
| Belgium | SDR | Specific-Trend | 2015-2019 | Weekly | 159.08 | 153.35 | 164.91 |
| Denmark | CDR | Specific-Average | 2015-2019 | Weekly | 5.83 | 2.53 | 9.55 |
| Denmark | CDR | Specific-Average with Trend | 2015-2019 | Weekly | -5.1 | -13.32 | 2.79 |
| Denmark | CDR | Harmonic with Trend | 2015-2019 | Weekly | -5.7 | -13.88 | 2.37 |
| Denmark | CDR | Specific-Trend | 2015-2019 | Weekly | -4.36 | -12.67 | 3.71 |
| Denmark | SDR | Specific-Average | 2015-2019 | Weekly | -42.3 | -45.76 | -38.76 |
| Denmark | SDR | Specific-Average with Trend | 2015-2019 | Weekly | -12.42 | -21.2 | -3.75 |
| Denmark | SDR | Harmonic with Trend | 2015-2019 | Weekly | -13.28 | -22.06 | -4.69 |
| Denmark | SDR | Specific-Trend | 2015-2019 | Weekly | -11.55 | -20.64 | -2.68 |
| Estonia | CDR | Specific-Average | 2015-2019 | Weekly | 23.51 | 15.2 | 31.55 |
| Estonia | CDR | Specific-Average with Trend | 2015-2019 | Weekly | 17.49 | -0.75 | 37.52 |
| Estonia | CDR | Harmonic with Trend | 2015-2019 | Weekly | 21.04 | 2.97 | 40.51 |
| Estonia | CDR | Specific-Trend | 2015-2019 | Weekly | 17.01 | -1.9 | 37.38 |
| Estonia | SDR | Specific-Average | 2015-2019 | Weekly | -38.14 | -46.74 | -29.44 |
| Estonia | SDR | Specific-Average with Trend | 2015-2019 | Weekly | 24.87 | 5.33 | 43.4 |
| Estonia | SDR | Harmonic with Trend | 2015-2019 | Weekly | 27.68 | 8.46 | 46.55 |
| Estonia | SDR | Specific-Trend | 2015-2019 | Weekly | 24.35 | 4.2 | 42.57 |
| France | CDR | Specific-Average | 2015-2019 | Weekly | 90.9 | 89.84 | 91.96 |
| France | CDR | Specific-Average with Trend | 2015-2019 | Weekly | 83.48 | 81.24 | 85.99 |
| France | CDR | Harmonic with Trend | 2015-2019 | Weekly | 85.83 | 83.59 | 88.36 |
| France | CDR | Specific-Trend | 2015-2019 | Weekly | 84.1 | 81.86 | 86.71 |
| France | SDR | Specific-Average | 2015-2019 | Weekly | 31.68 | 30.67 | 32.73 |
| France | SDR | Specific-Average with Trend | 2015-2019 | Weekly | 74.89 | 72.49 | 77.27 |
| France | SDR | Harmonic with Trend | 2015-2019 | Weekly | 76.63 | 74.24 | 78.94 |
| France | SDR | Specific-Trend | 2015-2019 | Weekly | 75.49 | 73.04 | 77.91 |
| Finland | CDR | Specific-Average | 2015-2019 | Weekly | 12.05 | 8.24 | 15.72 |
| Finland | CDR | Specific-Average with Trend | 2015-2019 | Weekly | -3.14 | -12.37 | 5.08 |
| Finland | CDR | Harmonic with Trend | 2015-2019 | Weekly | -0.59 | -9.69 | 7.71 |
| Finland | CDR | Specific-Trend | 2015-2019 | Weekly | -1.96 | -11.31 | 6.49 |
| Finland | SDR | Specific-Average | 2015-2019 | Weekly | -47.86 | -51.84 | -43.93 |
| Finland | SDR | Specific-Average with Trend | 2015-2019 | Weekly | -5.32 | -13.27 | 3.17 |
| Finland | SDR | Harmonic with Trend | 2015-2019 | Weekly | -3.14 | -11.08 | 5.26 |
| Finland | SDR | Specific-Trend | 2015-2019 | Weekly | -4.09 | -12.04 | 4.55 |
| Scotland | CDR | Specific-Average | 2015-2019 | Weekly | 108.94 | 105.07 | 112.54 |
| Scotland | CDR | Specific-Average with Trend | 2015-2019 | Weekly | 121.75 | 112.86 | 130.89 |
| Scotland | CDR | Harmonic with Trend | 2015-2019 | Weekly | 124.83 | 115.8 | 133.99 |
| Scotland | CDR | Specific-Trend | 2015-2019 | Weekly | 121.57 | 113.09 | 131.22 |
| Scotland | SDR | Specific-Average | 2015-2019 | Weekly | 65.38 | 61.46 | 69.82 |
| Scotland | SDR | Specific-Average with Trend | 2015-2019 | Weekly | 125.41 | 116.42 | 133.77 |
| Scotland | SDR | Harmonic with Trend | 2015-2019 | Weekly | 128.36 | 119.47 | 136.73 |
| Scotland | SDR | Specific-Trend | 2015-2019 | Weekly | 125.26 | 116.41 | 133.93 |
| England&Wales | CDR | Specific-Average | 2015-2019 | Weekly | 113.45 | 112.38 | 114.49 |
| England&Wales | CDR | Specific-Average with Trend | 2015-2019 | Weekly | 137.19 | 134.81 | 139.77 |
| England&Wales | CDR | Harmonic with Trend | 2015-2019 | Weekly | 140.11 | 137.77 | 142.67 |
| England&Wales | CDR | Specific-Trend | 2015-2019 | Weekly | 138.68 | 136.3 | 141.3 |
| England&Wales | SDR | Specific-Average | 2015-2019 | Weekly | 78.98 | 77.81 | 80.1 |
| England&Wales | SDR | Specific-Average with Trend | 2015-2019 | Weekly | 138.94 | 136.4 | 141.4 |
| England&Wales | SDR | Harmonic with Trend | 2015-2019 | Weekly | 141.8 | 139.28 | 144.25 |
| England&Wales | SDR | Specific-Trend | 2015-2019 | Weekly | 140.39 | 137.81 | 142.85 |
| Hungary | CDR | Specific-Average | 2015-2019 | Weekly | 145.16 | 141.79 | 148.24 |
| Hungary | CDR | Specific-Average with Trend | 2015-2019 | Weekly | 144.21 | 136.59 | 151.73 |
| Hungary | CDR | Harmonic with Trend | 2015-2019 | Weekly | 147.27 | 139.76 | 154.67 |
| Hungary | CDR | Specific-Trend | 2015-2019 | Weekly | 144.34 | 136.56 | 151.93 |
| Hungary | SDR | Specific-Average | 2015-2019 | Weekly | 69.34 | 66.27 | 72.76 |
| Hungary | SDR | Specific-Average with Trend | 2015-2019 | Weekly | 146.43 | 138.19 | 154.47 |
| Hungary | SDR | Harmonic with Trend | 2015-2019 | Weekly | 149.25 | 141.21 | 157.42 |
| Hungary | SDR | Specific-Trend | 2015-2019 | Weekly | 146.72 | 138.41 | 154.83 |
| Israel | CDR | Specific-Average | 2015-2019 | Weekly | 14.57 | 12.39 | 16.83 |
| Israel | CDR | Specific-Average with Trend | 2015-2019 | Weekly | 37.9 | 32.84 | 43.08 |
| Israel | CDR | Harmonic with Trend | 2015-2019 | Weekly | 39.15 | 34.16 | 44.55 |
| Israel | CDR | Specific-Trend | 2015-2019 | Weekly | 38.27 | 33.31 | 43.76 |
| Israel | SDR | Specific-Average | 2015-2019 | Weekly | -4.59 | -7.39 | -1.91 |
| Israel | SDR | Specific-Average with Trend | 2015-2019 | Weekly | 65.68 | 59.8 | 71.95 |
| Israel | SDR | Harmonic with Trend | 2015-2019 | Weekly | 67.74 | 61.75 | 73.74 |
| Israel | SDR | Specific-Trend | 2015-2019 | Weekly | 66.55 | 60.54 | 72.98 |
| Italy | CDR | Specific-Average | 2015-2019 | Weekly | 194.41 | 193.33 | 195.69 |
| Italy | CDR | Specific-Average with Trend | 2015-2019 | Weekly | 185.06 | 182.33 | 187.99 |
| Italy | CDR | Harmonic with Trend | 2015-2019 | Weekly | 189.54 | 186.85 | 192.5 |
| Italy | CDR | Specific-Trend | 2015-2019 | Weekly | 186.62 | 183.84 | 189.62 |
| Italy | SDR | Specific-Average | 2015-2019 | Weekly | 89.44 | 88.38 | 90.48 |
| Italy | SDR | Specific-Average with Trend | 2015-2019 | Weekly | 140.37 | 137.98 | 142.83 |
| Italy | SDR | Harmonic with Trend | 2015-2019 | Weekly | 143.55 | 141.19 | 146.07 |
| Italy | SDR | Specific-Trend | 2015-2019 | Weekly | 141.63 | 139.25 | 144.12 |
| R. of Korea | CDR | Specific-Average | 2015-2019 | Weekly | 29.94 | 29.09 | 30.87 |
| R. of Korea | CDR | Specific-Average with Trend | 2015-2019 | Weekly | 7.77 | 5.52 | 9.95 |
| R. of Korea | CDR | Harmonic with Trend | 2015-2019 | Weekly | 8.7 | 6.46 | 10.87 |
| R. of Korea | CDR | Specific-Trend | 2015-2019 | Weekly | 7.54 | 5.26 | 9.69 |
| R. of Korea | SDR | Specific-Average | 2015-2019 | Weekly | -70.9 | -71.98 | -69.63 |
| R. of Korea | SDR | Specific-Average with Trend | 2015-2019 | Weekly | 10.57 | 7.92 | 13.31 |
| R. of Korea | SDR | Harmonic with Trend | 2015-2019 | Weekly | 11.09 | 8.44 | 13.86 |
| R. of Korea | SDR | Specific-Trend | 2015-2019 | Weekly | 10.3 | 7.58 | 12.97 |
| Lithuania | CDR | Specific-Average | 2015-2019 | Weekly | 125.59 | 119.04 | 131.71 |
| Lithuania | CDR | Specific-Average with Trend | 2015-2019 | Weekly | 170.75 | 157.76 | 184.99 |
| Lithuania | CDR | Harmonic with Trend | 2015-2019 | Weekly | 176.03 | 163.04 | 190.58 |
| Lithuania | CDR | Specific-Trend | 2015-2019 | Weekly | 172.02 | 158.64 | 186.49 |
| Lithuania | SDR | Specific-Average | 2015-2019 | Weekly | 51.32 | 45.2 | 57.25 |
| Lithuania | SDR | Specific-Average with Trend | 2015-2019 | Weekly | 180.8 | 166.77 | 195.04 |
| Lithuania | SDR | Harmonic with Trend | 2015-2019 | Weekly | 185.43 | 171.06 | 199.99 |
| Lithuania | SDR | Specific-Trend | 2015-2019 | Weekly | 182.19 | 168.21 | 196.64 |
| Latvia | CDR | Specific-Average | 2015-2019 | Weekly | 27.83 | 20.58 | 35.57 |
| Latvia | CDR | Specific-Average with Trend | 2015-2019 | Weekly | 10.79 | -7.56 | 28.63 |
| Latvia | CDR | Harmonic with Trend | 2015-2019 | Weekly | 13.12 | -5.64 | 30.27 |
| Latvia | CDR | Specific-Trend | 2015-2019 | Weekly | 10.02 | -8.19 | 27.66 |
| Latvia | SDR | Specific-Average | 2015-2019 | Weekly | -40.73 | -47.9 | -33.29 |
| Latvia | SDR | Specific-Average with Trend | 2015-2019 | Weekly | 20.18 | 1.38 | 38.74 |
| Latvia | SDR | Harmonic with Trend | 2015-2019 | Weekly | 22.00 | 2.83 | 40.04 |
| Latvia | SDR | Specific-Trend | 2015-2019 | Weekly | 19.67 | 0.84 | 38.72 |
| Netherlands | CDR | Specific-Average | 2015-2019 | Weekly | 86.62 | 84.57 | 88.64 |
| Netherlands | CDR | Specific-Average with Trend | 2015-2019 | Weekly | 77.5 | 72.62 | 81.95 |
| Netherlands | CDR | Harmonic with Trend | 2015-2019 | Weekly | 78.39 | 73.48 | 82.83 |
| Netherlands | CDR | Specific-Trend | 2015-2019 | Weekly | 77.19 | 72.5 | 81.69 |
| Netherlands | SDR | Specific-Average | 2015-2019 | Weekly | 48.45 | 46.51 | 50.38 |
| Netherlands | SDR | Specific-Average with Trend | 2015-2019 | Weekly | 85.96 | 81.21 | 90.76 |
| Netherlands | SDR | Harmonic with Trend | 2015-2019 | Weekly | 86.68 | 82.01 | 91.63 |
| Netherlands | SDR | Specific-Trend | 2015-2019 | Weekly | 85.62 | 80.78 | 90.6 |
| Norway | CDR | Specific-Average | 2015-2019 | Weekly | -19.57 | -23.39 | -16.48 |
| Norway | CDR | Specific-Average with Trend | 2015-2019 | Weekly | -3.33 | -10.84 | 4.85 |
| Norway | CDR | Harmonic with Trend | 2015-2019 | Weekly | -1.85 | -9.53 | 6.41 |
| Norway | CDR | Specific-Trend | 2015-2019 | Weekly | -2.39 | -9.95 | 5.85 |
| Norway | SDR | Specific-Average | 2015-2019 | Weekly | -54.34 | -57.87 | -50.67 |
| Norway | SDR | Specific-Average with Trend | 2015-2019 | Weekly | -9.88 | -17.67 | -1.5 |
| Norway | SDR | Harmonic with Trend | 2015-2019 | Weekly | -8.26 | -15.97 | 0.02 |
| Norway | SDR | Specific-Trend | 2015-2019 | Weekly | -8.72 | -16.51 | -0.23 |
| New Zealand | CDR | Specific-Average | 2015-2019 | Weekly | -33.65 | -36.97 | -30.04 |
| New Zealand | CDR | Specific-Average with Trend | 2015-2019 | Weekly | -42.73 | -51.16 | -34.15 |
| New Zealand | CDR | Harmonic with Trend | 2015-2019 | Weekly | -42.92 | -51.24 | -34.63 |
| New Zealand | CDR | Specific-Trend | 2015-2019 | Weekly | -41.68 | -50.21 | -33.22 |
| New Zealand | SDR | Specific-Average | 2015-2019 | Weekly | -82.95 | -86.7 | -79.41 |
| New Zealand | SDR | Specific-Average with Trend | 2015-2019 | Weekly | -56.6 | -65.53 | -48.00 |
| New Zealand | SDR | Harmonic with Trend | 2015-2019 | Weekly | -57.24 | -66.26 | -48.67 |
| New Zealand | SDR | Specific-Trend | 2015-2019 | Weekly | -55.32 | -64.51 | -46.72 |
| Poland | CDR | Specific-Average | 2015-2019 | Weekly | 194.51 | 193.05 | 195.92 |
| Poland | CDR | Specific-Average with Trend | 2015-2019 | Weekly | 149.46 | 146.11 | 152.82 |
| Poland | CDR | Harmonic with Trend | 2015-2019 | Weekly | 151.05 | 147.69 | 154.4 |
| Poland | CDR | Specific-Trend | 2015-2019 | Weekly | 149.96 | 146.52 | 153.36 |
| Poland | SDR | Specific-Average | 2015-2019 | Weekly | 145.12 | 143.62 | 146.87 |
| Poland | SDR | Specific-Average with Trend | 2015-2019 | Weekly | 177.55 | 173.8 | 181.4 |
| Poland | SDR | Harmonic with Trend | 2015-2019 | Weekly | 178.75 | 175.05 | 182.64 |
| Poland | SDR | Specific-Trend | 2015-2019 | Weekly | 178.16 | 174.39 | 181.95 |
| Portugal | CDR | Specific-Average | 2015-2019 | Weekly | 131.3 | 128.33 | 134.25 |
| Portugal | CDR | Specific-Average with Trend | 2015-2019 | Weekly | 110.36 | 103.72 | 117.3 |
| Portugal | CDR | Harmonic with Trend | 2015-2019 | Weekly | 116.22 | 109.63 | 123.23 |
| Portugal | CDR | Specific-Trend | 2015-2019 | Weekly | 112.07 | 105.14 | 119.15 |
| Portugal | SDR | Specific-Average | 2015-2019 | Weekly | 55.15 | 52.47 | 57.91 |
| Portugal | SDR | Specific-Average with Trend | 2015-2019 | Weekly | 89.67 | 83.34 | 96.37 |
| Portugal | SDR | Harmonic with Trend | 2015-2019 | Weekly | 94.55 | 88.36 | 101.25 |
| Portugal | SDR | Specific-Trend | 2015-2019 | Weekly | 91.19 | 84.92 | 97.81 |
| Slovenia | CDR | Specific-Average | 2015-2019 | Weekly | 159.6 | 153.31 | 165.55 |
| Slovenia | CDR | Specific-Average with Trend | 2015-2019 | Weekly | 134.01 | 119.54 | 148.68 |
| Slovenia | CDR | Harmonic with Trend | 2015-2019 | Weekly | 136.37 | 122.17 | 150.98 |
| Slovenia | CDR | Specific-Trend | 2015-2019 | Weekly | 134.06 | 119.57 | 148.06 |
| Slovenia | SDR | Specific-Average | 2015-2019 | Weekly | 92.99 | 86.91 | 99.1 |
| Slovenia | SDR | Specific-Average with Trend | 2015-2019 | Weekly | 142.23 | 128.35 | 156.17 |
| Slovenia | SDR | Harmonic with Trend | 2015-2019 | Weekly | 144.12 | 130.68 | 158.35 |
| Slovenia | SDR | Specific-Trend | 2015-2019 | Weekly | 141.91 | 128.2 | 155.69 |
| Slovakia | CDR | Specific-Average | 2015-2019 | Weekly | 97.84 | 94.38 | 101.66 |
| Slovakia | CDR | Specific-Average with Trend | 2015-2019 | Weekly | 98.00 | 89.99 | 106.48 |
| Slovakia | CDR | Harmonic with Trend | 2015-2019 | Weekly | 100.4 | 92.38 | 108.59 |
| Slovakia | CDR | Specific-Trend | 2015-2019 | Weekly | 99.43 | 91.42 | 108.04 |
| Slovakia | SDR | Specific-Average | 2015-2019 | Weekly | 28.75 | 24.08 | 32.84 |
| Slovakia | SDR | Specific-Average with Trend | 2015-2019 | Weekly | 131.02 | 121.2 | 140.77 |
| Slovakia | SDR | Harmonic with Trend | 2015-2019 | Weekly | 133.39 | 123.67 | 143.3 |
| Slovakia | SDR | Specific-Trend | 2015-2019 | Weekly | 133.05 | 123.44 | 142.93 |
| Spain | CDR | Specific-Average | 2015-2019 | Weekly | 153.86 | 152.64 | 155.11 |
| Spain | CDR | Specific-Average with Trend | 2015-2019 | Weekly | 166.95 | 164.22 | 169.75 |
| Spain | CDR | Harmonic with Trend | 2015-2019 | Weekly | 170.46 | 167.69 | 173.34 |
| Spain | CDR | Specific-Trend | 2015-2019 | Weekly | 167.45 | 164.68 | 170.42 |
| Spain | SDR | Specific-Average | 2015-2019 | Weekly | 85.13 | 84.08 | 86.4 |
| Spain | SDR | Specific-Average with Trend | 2015-2019 | Weekly | 146.65 | 143.82 | 149.19 |
| Spain | SDR | Harmonic with Trend | 2015-2019 | Weekly | 149.5 | 146.73 | 152.09 |
| Spain | SDR | Specific-Trend | 2015-2019 | Weekly | 147.15 | 144.34 | 149.79 |
| Sweden | CDR | Specific-Average | 2015-2019 | Weekly | 13.28 | 10.58 | 15.98 |
| Sweden | CDR | Specific-Average with Trend | 2015-2019 | Weekly | 53.71 | 47.96 | 60.06 |
| Sweden | CDR | Harmonic with Trend | 2015-2019 | Weekly | 54.57 | 48.75 | 60.93 |
| Sweden | CDR | Specific-Trend | 2015-2019 | Weekly | 54.28 | 48.36 | 60.51 |
| Sweden | SDR | Specific-Average | 2015-2019 | Weekly | -9.59 | -12.15 | -6.91 |
| Sweden | SDR | Specific-Average with Trend | 2015-2019 | Weekly | 40.6 | 35.22 | 46.95 |
| Sweden | SDR | Harmonic with Trend | 2015-2019 | Weekly | 41.43 | 36.02 | 47.74 |
| Sweden | SDR | Specific-Trend | 2015-2019 | Weekly | 41.18 | 35.65 | 47.52 |
| Switzerland | CDR | Specific-Average | 2015-2019 | Weekly | 92.31 | 89.52 | 95.05 |
| Switzerland | CDR | Specific-Average with Trend | 2015-2019 | Weekly | 113.3 | 107.03 | 119.46 |
| Switzerland | CDR | Harmonic with Trend | 2015-2019 | Weekly | 114.25 | 107.94 | 120.33 |
| Switzerland | CDR | Specific-Trend | 2015-2019 | Weekly | 114.07 | 107.51 | 120.08 |
| Switzerland | SDR | Specific-Average | 2015-2019 | Weekly | 45.94 | 43.3 | 48.61 |
| Switzerland | SDR | Specific-Average with Trend | 2015-2019 | Weekly | 106.65 | 100.57 | 112.81 |
| Switzerland | SDR | Harmonic with Trend | 2015-2019 | Weekly | 107.29 | 101.49 | 113.48 |
| Switzerland | SDR | Specific-Trend | 2015-2019 | Weekly | 107.43 | 101.24 | 113.56 |
| Taiwan | CDR | Specific-Average | 2015-2019 | Weekly | -13.85 | -15.31 | -12.28 |
| Taiwan | CDR | Specific-Average with Trend | 2015-2019 | Weekly | -40.75 | -44.44 | -37.11 |
| Taiwan | CDR | Harmonic with Trend | 2015-2019 | Weekly | -40.83 | -44.6 | -37.11 |
| Taiwan | CDR | Specific-Trend | 2015-2019 | Weekly | -40.75 | -44.44 | -37.11 |
| Taiwan | SDR | Specific-Average | 2015-2019 | Weekly | -108.45 | -110.13 | -106.68 |
| Taiwan | SDR | Specific-Average with Trend | 2015-2019 | Weekly | -53.45 | -57.51 | -49.6 |
| Taiwan | SDR | Harmonic with Trend | 2015-2019 | Weekly | -54.51 | -58.61 | -50.53 |
| Taiwan | SDR | Specific-Trend | 2015-2019 | Weekly | -53.45 | -57.51 | -49.6 |
| U.S.A. | CDR | Specific-Average | 2015-2019 | Weekly | 158.1 | 157.66 | 158.58 |
| U.S.A. | CDR | Specific-Average with Trend | 2015-2019 | Weekly | 138.13 | 137.04 | 139.15 |
| U.S.A. | CDR | Harmonic with Trend | 2015-2019 | Weekly | 139.25 | 138.15 | 140.3 |
| U.S.A. | CDR | Specific-Trend | 2015-2019 | Weekly | 137.76 | 136.65 | 138.8 |
| U.S.A. | SDR | Specific-Average | 2015-2019 | Weekly | 120.27 | 119.76 | 120.74 |
| U.S.A. | SDR | Specific-Average with Trend | 2015-2019 | Weekly | 153.98 | 152.9 | 155.16 |
| U.S.A. | SDR | Harmonic with Trend | 2015-2019 | Weekly | 155.01 | 153.93 | 156.24 |
| U.S.A. | SDR | Specific-Trend | 2015-2019 | Weekly | 153.62 | 152.54 | 154.85 |

**Source**: Jdanov et al. (2021) and European Commission (2013).

**Table 2B:** Excess mortality rates (per 100,000 persons) for Scenarios 2, 6, 9-12 by country, 2020.

| **Country** | **Index** | **Method** | **Reference Period** | **Time Unit** | **Excess Mortality** | **Confidence Interval** | |
| --- | --- | --- | --- | --- | --- | --- | --- |
|  |  |  |  |  |  | **Lower limit** | **Upper limit** |
| Austria | CDR | Specific-Average with Trend | 2017-2019 | Weekly | 70.16 | 60.35 | 79.96 |
| Austria | CDR | Specific-Average with Trend | 2015-2019 | Weekly | 68.45 | 62.05 | 75.56 |
| Austria | CDR | Specific-Average with Trend | 2010-2019 | Weekly | 57.91 | 53.48 | 61.9 |
| Austria | SDR | Specific-Average with Trend | 2017-2019 | Weekly | 64.18 | 54.01 | 74.98 |
| Austria | SDR | Specific-Average with Trend | 2015-2019 | Weekly | 60.6 | 53.5 | 68.03 |
| Austria | SDR | Specific-Average with Trend | 2010-2019 | Weekly | 54.91 | 50.54 | 59.34 |
| Belgium | CDR | Specific-Average with Trend | 2017-2019 | Weekly | 183.37 | 175.13 | 191.28 |
| Belgium | CDR | Specific-Average with Trend | 2015-2019 | Weekly | 169.25 | 163.54 | 175.09 |
| Belgium | CDR | Specific-Average with Trend | 2010-2019 | Weekly | 150.96 | 147.19 | 154.79 |
| Belgium | SDR | Specific-Average with Trend | 2017-2019 | Weekly | 170.89 | 163.02 | 179.42 |
| Belgium | SDR | Specific-Average with Trend | 2015-2019 | Weekly | 159.32 | 153.69 | 165.04 |
| Belgium | SDR | Specific-Average with Trend | 2010-2019 | Weekly | 142.29 | 138.44 | 145.88 |
| Denmark | CDR | Specific-Average with Trend | 2017-2019 | Weekly | -2.46 | -15.29 | 10.28 |
| Denmark | CDR | Specific-Average with Trend | 2015-2019 | Weekly | -5.1 | -13.32 | 2.79 |
| Denmark | CDR | Specific-Average with Trend | 2010-2019 | Weekly | 16.23 | 10.38 | 21.31 |
| Denmark | SDR | Specific-Average with Trend | 2017-2019 | Weekly | -8.37 | -20.17 | 3.21 |
| Denmark | SDR | Specific-Average with Trend | 2015-2019 | Weekly | -12.42 | -21.2 | -3.75 |
| Denmark | SDR | Specific-Average with Trend | 2010-2019 | Weekly | 12.42 | 6.64 | 18.06 |
| Estonia | CDR | Specific-Average with Trend | 2017-2019 | Weekly | 37.69 | 11.4 | 66.01 |
| Estonia | CDR | Specific-Average with Trend | 2015-2019 | Weekly | 17.49 | -0.75 | 37.52 |
| Estonia | CDR | Specific-Average with Trend | 2010-2019 | Weekly | 20.18 | 7.41 | 32.46 |
| Estonia | SDR | Specific-Average with Trend | 2017-2019 | Weekly | 36.01 | 5.64 | 62.03 |
| Estonia | SDR | Specific-Average with Trend | 2015-2019 | Weekly | 24.87 | 5.33 | 43.4 |
| Estonia | SDR | Specific-Average with Trend | 2010-2019 | Weekly | 39.27 | 26.59 | 51.41 |
| France | CDR | Specific-Average with Trend | 2017-2019 | Weekly | 99.56 | 95.94 | 103.15 |
| France | CDR | Specific-Average with Trend | 2015-2019 | Weekly | 83.48 | 81.24 | 85.99 |
| France | CDR | Specific-Average with Trend | 2010-2019 | Weekly | 72.77 | 71.11 | 74.29 |
| France | SDR | Specific-Average with Trend | 2017-2019 | Weekly | 85.29 | 82.1 | 88.31 |
| France | SDR | Specific-Average with Trend | 2015-2019 | Weekly | 74.89 | 72.49 | 77.27 |
| France | SDR | Specific-Average with Trend | 2010-2019 | Weekly | 66.52 | 64.99 | 68.01 |
| Finland | CDR | Specific-Average with Trend | 2017-2019 | Weekly | 7.51 | -4.06 | 20.14 |
| Finland | CDR | Specific-Average with Trend | 2015-2019 | Weekly | -3.14 | -12.37 | 5.08 |
| Finland | CDR | Specific-Average with Trend | 2010-2019 | Weekly | -1.4 | -6.73 | 4.29 |
| Finland | SDR | Specific-Average with Trend | 2017-2019 | Weekly | 5.17 | -6.63 | 17.14 |
| Finland | SDR | Specific-Average with Trend | 2015-2019 | Weekly | -5.32 | -13.27 | 3.17 |
| Finland | SDR | Specific-Average with Trend | 2010-2019 | Weekly | -3.22 | -8.52 | 2.69 |
| Scotland | CDR | Specific-Average with Trend | 2017-2019 | Weekly | 136.74 | 124.13 | 150.05 |
| Scotland | CDR | Specific-Average with Trend | 2015-2019 | Weekly | 121.75 | 112.86 | 130.89 |
| Scotland | CDR | Specific-Average with Trend | 2010-2019 | Weekly | 99.55 | 93.57 | 105.78 |
| Scotland | SDR | Specific-Average with Trend | 2017-2019 | Weekly | 142.05 | 129.28 | 155.98 |
| Scotland | SDR | Specific-Average with Trend | 2015-2019 | Weekly | 125.41 | 116.42 | 133.77 |
| Scotland | SDR | Specific-Average with Trend | 2010-2019 | Weekly | 99.89 | 93.9 | 105.48 |
| England&Wales | CDR | Specific-Average with Trend | 2017-2019 | Weekly | 154.56 | 150.93 | 157.83 |
| England&Wales | CDR | Specific-Average with Trend | 2015-2019 | Weekly | 137.19 | 134.81 | 139.77 |
| England&Wales | CDR | Specific-Average with Trend | 2010-2019 | Weekly | 109.8 | 108.17 | 111.36 |
| England&Wales | SDR | Specific-Average with Trend | 2017-2019 | Weekly | 156.9 | 153.17 | 160.64 |
| England&Wales | SDR | Specific-Average with Trend | 2015-2019 | Weekly | 138.94 | 136.4 | 141.4 |
| England&Wales | SDR | Specific-Average with Trend | 2010-2019 | Weekly | 109.34 | 107.77 | 111.06 |
| Hungary | CDR | Specific-Average with Trend | 2017-2019 | Weekly | 148.65 | 137.79 | 158.87 |
| Hungary | CDR | Specific-Average with Trend | 2015-2019 | Weekly | 144.21 | 136.59 | 151.73 |
| Hungary | CDR | Specific-Average with Trend | 2010-2019 | Weekly | 143.18 | 138.48 | 147.9 |
| Hungary | SDR | Specific-Average with Trend | 2017-2019 | Weekly | 149.74 | 138.71 | 161.98 |
| Hungary | SDR | Specific-Average with Trend | 2015-2019 | Weekly | 146.43 | 138.19 | 154.47 |
| Hungary | SDR | Specific-Average with Trend | 2010-2019 | Weekly | 145.84 | 140.73 | 150.75 |
| Israel | CDR | Specific-Average with Trend | 2017-2019 | Weekly | 40.51 | 34.23 | 47.41 |
| Israel | CDR | Specific-Average with Trend | 2015-2019 | Weekly | 37.9 | 32.84 | 43.08 |
| Israel | CDR | Specific-Average with Trend | 2010-2019 | Weekly | 24.03 | 20.7 | 27.12 |
| Israel | SDR | Specific-Average with Trend | 2017-2019 | Weekly | 68.86 | 59.85 | 77.76 |
| Israel | SDR | Specific-Average with Trend | 2015-2019 | Weekly | 65.68 | 59.8 | 71.95 |
| Israel | SDR | Specific-Average with Trend | 2010-2019 | Weekly | 41.58 | 37.77 | 45.79 |
| Italy | CDR | Specific-Average with Trend | 2017-2019 | Weekly | 204.38 | 200.33 | 208.13 |
| Italy | CDR | Specific-Average with Trend | 2015-2019 | Weekly | 185.06 | 182.33 | 187.99 |
| Italy | SDR | Specific-Average with Trend | 2017-2019 | Weekly | 155.19 | 151.64 | 158.88 |
| Italy | SDR | Specific-Average with Trend | 2015-2019 | Weekly | 140.37 | 137.98 | 142.83 |
| R. of Korea | CDR | Specific-Average with Trend | 2017-2019 | Weekly | 16.51 | 13.37 | 19.67 |
| R. of Korea | CDR | Specific-Average with Trend | 2015-2019 | Weekly | 7.77 | 5.52 | 9.95 |
| R. of Korea | CDR | Specific-Average with Trend | 2010-2019 | Weekly | 10.12 | 8.75 | 11.59 |
| R. of Korea | SDR | Specific-Average with Trend | 2017-2019 | Weekly | 26.73 | 23.3 | 30.5 |
| R. of Korea | SDR | Specific-Average with Trend | 2015-2019 | Weekly | 10.57 | 7.92 | 13.31 |
| R. of Korea | SDR | Specific-Average with Trend | 2010-2019 | Weekly | 12.77 | 11.05 | 14.48 |
| Lithuania | CDR | Specific-Average with Trend | 2017-2019 | Weekly | 189.09 | 168.26 | 210.42 |
| Lithuania | CDR | Specific-Average with Trend | 2015-2019 | Weekly | 170.75 | 157.76 | 184.99 |
| Lithuania | CDR | Specific-Average with Trend | 2010-2019 | Weekly | 116.77 | 107.49 | 126.47 |
| Lithuania | SDR | Specific-Average with Trend | 2017-2019 | Weekly | 189.42 | 168.57 | 209.57 |
| Lithuania | SDR | Specific-Average with Trend | 2015-2019 | Weekly | 180.8 | 166.77 | 195.04 |
| Lithuania | SDR | Specific-Average with Trend | 2010-2019 | Weekly | 139.46 | 130.52 | 148.88 |
| Latvia | CDR | Specific-Average with Trend | 2017-2019 | Weekly | 49.19 | 22.36 | 74.74 |
| Latvia | CDR | Specific-Average with Trend | 2015-2019 | Weekly | 10.79 | -7.56 | 28.63 |
| Latvia | CDR | Specific-Average with Trend | 2010-2019 | Weekly | 7.48 | -4.24 | 18.82 |
| Latvia | SDR | Specific-Average with Trend | 2017-2019 | Weekly | 53.51 | 28.67 | 78.78 |
| Latvia | SDR | Specific-Average with Trend | 2015-2019 | Weekly | 20.18 | 1.38 | 38.74 |
| Latvia | SDR | Specific-Average with Trend | 2010-2019 | Weekly | 31.72 | 20.14 | 44.62 |
| Netherlands | CDR | Specific-Average with Trend | 2017-2019 | Weekly | 84.1 | 76.7 | 91.09 |
| Netherlands | CDR | Specific-Average with Trend | 2015-2019 | Weekly | 77.5 | 72.62 | 81.95 |
| Netherlands | CDR | Specific-Average with Trend | 2010-2019 | Weekly | 65.18 | 62.23 | 67.99 |
| Netherlands | SDR | Specific-Average with Trend | 2017-2019 | Weekly | 92.86 | 85.82 | 100.49 |
| Netherlands | SDR | Specific-Average with Trend | 2015-2019 | Weekly | 85.96 | 81.21 | 90.76 |
| Netherlands | SDR | Specific-Average with Trend | 2010-2019 | Weekly | 78.2 | 74.92 | 81.47 |
| Norway | CDR | Specific-Average with Trend | 2017-2019 | Weekly | -1.56 | -12.91 | 9.43 |
| Norway | CDR | Specific-Average with Trend | 2015-2019 | Weekly | -3.33 | -10.84 | 4.85 |
| Norway | CDR | Specific-Average with Trend | 2010-2019 | Weekly | 10.94 | 5.71 | 15.91 |
| Norway | SDR | Specific-Average with Trend | 2017-2019 | Weekly | -6.29 | -17.63 | 5.2 |
| Norway | SDR | Specific-Average with Trend | 2015-2019 | Weekly | -9.88 | -17.67 | -1.5 |
| Norway | SDR | Specific-Average with Trend | 2010-2019 | Weekly | -2.17 | -7.82 | 3.32 |
| New Zealand | CDR | Specific-Average with Trend | 2017-2019 | Weekly | -33.84 | -45.92 | -22.55 |
| New Zealand | CDR | Specific-Average with Trend | 2015-2019 | Weekly | -42.73 | -51.16 | -34.15 |
| New Zealand | SDR | Specific-Average with Trend | 2017-2019 | Weekly | -44.76 | -58.3 | -31.98 |
| New Zealand | SDR | Specific-Average with Trend | 2015-2019 | Weekly | -56.6 | -65.53 | -48.00 |
| Poland | CDR | Specific-Average with Trend | 2017-2019 | Weekly | 157.33 | 152.23 | 162.16 |
| Poland | CDR | Specific-Average with Trend | 2015-2019 | Weekly | 149.46 | 146.11 | 152.82 |
| Poland | CDR | Specific-Average with Trend | 2010-2019 | Weekly | 163.53 | 161.27 | 165.67 |
| Poland | SDR | Specific-Average with Trend | 2017-2019 | Weekly | 184.5 | 179.51 | 189.88 |
| Poland | SDR | Specific-Average with Trend | 2015-2019 | Weekly | 177.55 | 173.8 | 181.4 |
| Poland | SDR | Specific-Average with Trend | 2010-2019 | Weekly | 198.56 | 196.27 | 200.93 |
| Portugal | CDR | Specific-Average with Trend | 2017-2019 | Weekly | 122.33 | 112.86 | 132.48 |
| Portugal | CDR | Specific-Average with Trend | 2015-2019 | Weekly | 110.36 | 103.72 | 117.3 |
| Portugal | CDR | Specific-Average with Trend | 2010-2019 | Weekly | 101.78 | 97.25 | 106.37 |
| Portugal | SDR | Specific-Average with Trend | 2017-2019 | Weekly | 95.37 | 87.04 | 104.38 |
| Portugal | SDR | Specific-Average with Trend | 2015-2019 | Weekly | 89.67 | 83.34 | 96.37 |
| Portugal | SDR | Specific-Average with Trend | 2010-2019 | Weekly | 95.41 | 91.3 | 99.13 |
| Slovenia | CDR | Specific-Average with Trend | 2017-2019 | Weekly | 153.56 | 133.27 | 175.51 |
| Slovenia | CDR | Specific-Average with Trend | 2015-2019 | Weekly | 134.01 | 119.54 | 148.68 |
| Slovenia | CDR | Specific-Average with Trend | 2010-2019 | Weekly | 132.67 | 123.36 | 141.68 |
| Slovenia | SDR | Specific-Average with Trend | 2017-2019 | Weekly | 157.57 | 133.66 | 176.91 |
| Slovenia | SDR | Specific-Average with Trend | 2015-2019 | Weekly | 142.23 | 128.35 | 156.17 |
| Slovenia | SDR | Specific-Average with Trend | 2010-2019 | Weekly | 145.07 | 135.69 | 153.67 |
| Slovakia | CDR | Specific-Average with Trend | 2017-2019 | Weekly | 109.95 | 97.12 | 123.03 |
| Slovakia | CDR | Specific-Average with Trend | 2015-2019 | Weekly | 98.00 | 89.99 | 106.48 |
| Slovakia | CDR | Specific-Average with Trend | 2010-2019 | Weekly | 97.78 | 92.31 | 103.3 |
| Slovakia | SDR | Specific-Average with Trend | 2017-2019 | Weekly | 154.25 | 140.55 | 168.62 |
| Slovakia | SDR | Specific-Average with Trend | 2015-2019 | Weekly | 131.02 | 121.2 | 140.77 |
| Slovakia | SDR | Specific-Average with Trend | 2010-2019 | Weekly | 125.32 | 118.16 | 132.4 |
| Spain | CDR | Specific-Average with Trend | 2017-2019 | Weekly | 191.1 | 187.07 | 194.88 |
| Spain | CDR | Specific-Average with Trend | 2015-2019 | Weekly | 166.95 | 164.22 | 169.75 |
| Spain | CDR | Specific-Average with Trend | 2010-2019 | Weekly | 133.32 | 131.47 | 135.21 |
| Spain | SDR | Specific-Average with Trend | 2017-2019 | Weekly | 163.2 | 159.39 | 167.48 |
| Spain | SDR | Specific-Average with Trend | 2015-2019 | Weekly | 146.65 | 143.82 | 149.19 |
| Spain | SDR | Specific-Average with Trend | 2010-2019 | Weekly | 127.05 | 125.31 | 128.78 |
| Sweden | CDR | Specific-Average with Trend | 2017-2019 | Weekly | 75.17 | 66.94 | 83.63 |
| Sweden | CDR | Specific-Average with Trend | 2015-2019 | Weekly | 53.71 | 47.96 | 60.06 |
| Sweden | CDR | Specific-Average with Trend | 2010-2019 | Weekly | 42.86 | 38.99 | 46.95 |
| Sweden | SDR | Specific-Average with Trend | 2017-2019 | Weekly | 64.79 | 55.66 | 73.49 |
| Sweden | SDR | Specific-Average with Trend | 2015-2019 | Weekly | 40.6 | 35.22 | 46.95 |
| Sweden | SDR | Specific-Average with Trend | 2010-2019 | Weekly | 31.18 | 27.46 | 34.83 |
| Switzerland | CDR | Specific-Average with Trend | 2017-2019 | Weekly | 115.24 | 106.38 | 124.21 |
| Switzerland | CDR | Specific-Average with Trend | 2015-2019 | Weekly | 113.3 | 107.03 | 119.46 |
| Switzerland | CDR | Specific-Average with Trend | 2010-2019 | Weekly | 100.43 | 96.45 | 104.43 |
| Switzerland | SDR | Specific-Average with Trend | 2017-2019 | Weekly | 107.91 | 99.09 | 116.9 |
| Switzerland | SDR | Specific-Average with Trend | 2015-2019 | Weekly | 106.65 | 100.57 | 112.81 |
| Switzerland | SDR | Specific-Average with Trend | 2010-2019 | Weekly | 88.63 | 84.68 | 93.00 |
| Taiwan | CDR | Specific-Average with Trend | 2017-2019 | Weekly | -34.96 | -39.99 | -29.89 |
| Taiwan | CDR | Specific-Average with Trend | 2015-2019 | Weekly | -40.75 | -44.44 | -37.11 |
| Taiwan | CDR | Specific-Average with Trend | 2010-2019 | Weekly | -50.31 | -52.7 | -48.09 |
| Taiwan | SDR | Specific-Average with Trend | 2017-2019 | Weekly | -41.54 | -47.88 | -35.99 |
| Taiwan | SDR | Specific-Average with Trend | 2015-2019 | Weekly | -53.45 | -57.51 | -49.6 |
| Taiwan | SDR | Specific-Average with Trend | 2010-2019 | Weekly | -65.38 | -68.32 | -62.54 |
| U.S.A. | CDR | Specific-Average with Trend | 2017-2019 | Weekly | 144.63 | 143.14 | 146.07 |
| U.S.A. | CDR | Specific-Average with Trend | 2015-2019 | Weekly | 138.13 | 137.04 | 139.15 |
| U.S.A. | SDR | Specific-Average with Trend | 2017-2019 | Weekly | 163.51 | 161.92 | 165.25 |
| U.S.A. | SDR | Specific-Average with Trend | 2015-2019 | Weekly | 153.98 | 152.9 | 155.16 |

**Source**: Jdanov et al. (2021) and European Commission (2013).

**Table 3B:** Excess mortality rates (per 100,000 persons) for Scenarios 13-16 by country, 2020.

| **Country** | **Index** | **Method** | **Reference Period** | **Time Unit** | **Excess Mortality** | **Confidence Interval** | |
| --- | --- | --- | --- | --- | --- | --- | --- |
|  |  |  |  |  |  | **Lower limit** | **Upper limit** |
| Austria | CDR | Harmonic with Trend | 2015-2019 | Weekly | 72.09 | 65.6 | 79.11 |
| Austria | CDR | Harmonic with Trend | 2015-2019 | Monthly | 71.71 | 65.32 | 78.64 |
| Austria | SDR | Harmonic with Trend | 2015-2019 | Weekly | 64.01 | 57.05 | 71.18 |
| Austria | SDR | Harmonic with Trend | 2015-2019 | Monthly | 63.95 | 56.95 | 70.31 |
| Belgium | CDR | Harmonic with Trend | 2015-2019 | Weekly | 171.47 | 165.86 | 177.34 |
| Belgium | CDR | Harmonic with Trend | 2015-2019 | Monthly | 171.54 | 165.61 | 177.15 |
| Belgium | SDR | Harmonic with Trend | 2015-2019 | Weekly | 161.19 | 155.67 | 166.97 |
| Belgium | SDR | Harmonic with Trend | 2015-2019 | Monthly | 161.29 | 155.21 | 167.3 |
| Denmark | CDR | Harmonic with Trend | 2015-2019 | Weekly | -4.54 | -12.71 | 3.43 |
| Denmark | CDR | Harmonic with Trend | 2015-2019 | Monthly | -5.16 | -13.66 | 3.12 |
| Denmark | SDR | Harmonic with Trend | 2015-2019 | Weekly | -12.19 | -20.89 | -3.55 |
| Denmark | SDR | Harmonic with Trend | 2015-2019 | Monthly | -13.2 | -22.52 | -4.3 |
| Estonia | CDR | Harmonic with Trend | 2015-2019 | Weekly | 22.74 | 4.66 | 42.3 |
| Estonia | CDR | Harmonic with Trend | 2015-2019 | Monthly | 23.39 | 3.83 | 41.41 |
| Estonia | SDR | Harmonic with Trend | 2015-2019 | Weekly | 29.14 | 10.07 | 48.07 |
| Estonia | SDR | Harmonic with Trend | 2015-2019 | Monthly | 29.81 | 10.26 | 48.45 |
| France | CDR | Harmonic with Trend | 2015-2019 | Weekly | 87.97 | 85.76 | 90.51 |
| France | CDR | Harmonic with Trend | 2015-2019 | Monthly | 88.16 | 86.12 | 90.64 |
| France | SDR | Harmonic with Trend | 2015-2019 | Weekly | 78.42 | 76.07 | 80.7 |
| France | SDR | Harmonic with Trend | 2015-2019 | Monthly | 78.7 | 76.48 | 81.08 |
| Finland | CDR | Harmonic with Trend | 2015-2019 | Weekly | 0.83 | -8.17 | 9.15 |
| Finland | CDR | Harmonic with Trend | 2015-2019 | Monthly | 1.78 | -6.22 | 10.4 |
| Finland | SDR | Harmonic with Trend | 2015-2019 | Weekly | -1.85 | -9.79 | 6.46 |
| Finland | SDR | Harmonic with Trend | 2015-2019 | Monthly | -0.93 | -9.08 | 7.51 |
| Scotland | CDR | Harmonic with Trend | 2015-2019 | Weekly | 127.7 | 118.69 | 136.71 |
| Scotland | CDR | Harmonic with Trend | 2015-2019 | Monthly | 125.8 | 116.81 | 134.2 |
| Scotland | SDR | Harmonic with Trend | 2015-2019 | Weekly | 131.3 | 122.43 | 139.73 |
| Scotland | SDR | Harmonic with Trend | 2015-2019 | Monthly | 128.95 | 120.31 | 137.96 |
| England&Wales | CDR | Harmonic with Trend | 2015-2019 | Weekly | 142.25 | 139.91 | 144.81 |
| England&Wales | CDR | Harmonic with Trend | 2015-2019 | Monthly | 138.95 | 136.56 | 141.4 |
| England&Wales | SDR | Harmonic with Trend | 2015-2019 | Weekly | 143.97 | 141.42 | 146.42 |
| England&Wales | SDR | Harmonic with Trend | 2015-2019 | Monthly | 140.4 | 137.95 | 142.81 |
| Hungary | CDR | Harmonic with Trend | 2015-2019 | Weekly | 150.09 | 142.64 | 157.56 |
| Hungary | CDR | Harmonic with Trend | 2015-2019 | Monthly | 149.36 | 142.12 | 156.99 |
| Hungary | SDR | Harmonic with Trend | 2015-2019 | Weekly | 152.07 | 144.04 | 160.23 |
| Hungary | SDR | Harmonic with Trend | 2015-2019 | Monthly | 151.88 | 144.35 | 159.47 |
| Israel | CDR | Harmonic with Trend | 2015-2019 | Weekly | 40.51 | 35.51 | 45.87 |
| Israel | CDR | Harmonic with Trend | 2015-2019 | Monthly | 41.19 | 36.45 | 46.14 |
| Israel | SDR | Harmonic with Trend | 2015-2019 | Weekly | 69.96 | 63.9 | 75.92 |
| Israel | SDR | Harmonic with Trend | 2015-2019 | Monthly | 70.85 | 64.45 | 77.13 |
| Italy | CDR | Harmonic with Trend | 2015-2019 | Weekly | 192.48 | 189.81 | 195.42 |
| Italy | CDR | Harmonic with Trend | 2015-2019 | Monthly | 192.61 | 190.1 | 195.45 |
| Italy | SDR | Harmonic with Trend | 2015-2019 | Weekly | 145.77 | 143.41 | 148.3 |
| Italy | SDR | Harmonic with Trend | 2015-2019 | Monthly | 146.1 | 143.85 | 148.58 |
| R. of Korea | CDR | Harmonic with Trend | 2015-2019 | Weekly | 9.56 | 7.32 | 11.74 |
| R. of Korea | CDR | Harmonic with Trend | 2015-2019 | Monthly | 9.45 | 7.49 | 11.35 |
| R. of Korea | SDR | Harmonic with Trend | 2015-2019 | Weekly | 12.04 | 9.41 | 14.79 |
| R. of Korea | SDR | Harmonic with Trend | 2015-2019 | Monthly | 12.22 | 9.69 | 14.57 |
| Lithuania | CDR | Harmonic with Trend | 2015-2019 | Weekly | 178.27 | 165.14 | 192.78 |
| Lithuania | CDR | Harmonic with Trend | 2015-2019 | Monthly | 180.59 | 166.07 | 195.13 |
| Lithuania | SDR | Harmonic with Trend | 2015-2019 | Weekly | 187.47 | 173.2 | 201.83 |
| Lithuania | SDR | Harmonic with Trend | 2015-2019 | Monthly | 189.43 | 175.31 | 203.55 |
| Latvia | CDR | Harmonic with Trend | 2015-2019 | Weekly | 15.26 | -3.57 | 32.24 |
| Latvia | CDR | Harmonic with Trend | 2015-2019 | Monthly | 16.48 | -1.89 | 34.29 |
| Latvia | SDR | Harmonic with Trend | 2015-2019 | Weekly | 23.91 | 5.06 | 41.92 |
| Latvia | SDR | Harmonic with Trend | 2015-2019 | Monthly | 25.17 | 8.53 | 43.35 |
| Netherlands | CDR | Harmonic with Trend | 2015-2019 | Weekly | 80.15 | 75.25 | 84.54 |
| Netherlands | CDR | Harmonic with Trend | 2015-2019 | Monthly | 80.00 | 75.23 | 84.38 |
| Netherlands | SDR | Harmonic with Trend | 2015-2019 | Weekly | 88.46 | 83.78 | 93.36 |
| Netherlands | SDR | Harmonic with Trend | 2015-2019 | Monthly | 88.55 | 83.92 | 93.24 |
| Norway | CDR | Harmonic with Trend | 2015-2019 | Weekly | -0.25 | -7.83 | 7.95 |
| Norway | CDR | Harmonic with Trend | 2015-2019 | Monthly | 0.19 | -7.47 | 7.83 |
| Norway | SDR | Harmonic with Trend | 2015-2019 | Weekly | -6.51 | -14.18 | 1.8 |
| Norway | SDR | Harmonic with Trend | 2015-2019 | Monthly | -6.03 | -14.2 | 1.62 |
| New Zealand | CDR | Harmonic with Trend | 2015-2019 | Weekly | -43.87 | -52.33 | -35.68 |
| New Zealand | CDR | Harmonic with Trend | 2015-2019 | Monthly | -41.95 | -50.05 | -35.00 |
| New Zealand | SDR | Harmonic with Trend | 2015-2019 | Weekly | -58.64 | -67.6 | -50.03 |
| New Zealand | SDR | Harmonic with Trend | 2015-2019 | Monthly | -56.42 | -65.55 | -47.86 |
| Poland | CDR | Harmonic with Trend | 2015-2019 | Weekly | 152.72 | 149.4 | 156.04 |
| Poland | CDR | Harmonic with Trend | 2015-2019 | Monthly | 153.23 | 149.97 | 156.78 |
| Poland | SDR | Harmonic with Trend | 2015-2019 | Weekly | 180.47 | 176.78 | 184.34 |
| Poland | SDR | Harmonic with Trend | 2015-2019 | Monthly | 180.98 | 177.5 | 184.65 |
| Portugal | CDR | Harmonic with Trend | 2015-2019 | Weekly | 120.49 | 113.92 | 127.52 |
| Portugal | CDR | Harmonic with Trend | 2015-2019 | Monthly | 121.69 | 115.12 | 127.91 |
| Portugal | SDR | Harmonic with Trend | 2015-2019 | Weekly | 98.3 | 92.11 | 105.06 |
| Portugal | SDR | Harmonic with Trend | 2015-2019 | Monthly | 99.79 | 93.58 | 106.13 |
| Slovenia | CDR | Harmonic with Trend | 2015-2019 | Weekly | 138.56 | 124.09 | 152.93 |
| Slovenia | CDR | Harmonic with Trend | 2015-2019 | Monthly | 138.49 | 124.8 | 151.91 |
| Slovenia | SDR | Harmonic with Trend | 2015-2019 | Weekly | 146.05 | 132.65 | 160.18 |
| Slovenia | SDR | Harmonic with Trend | 2015-2019 | Monthly | 146.63 | 133.77 | 160.93 |
| Slovakia | CDR | Harmonic with Trend | 2015-2019 | Weekly | 101.88 | 93.88 | 110.12 |
| Slovakia | CDR | Harmonic with Trend | 2015-2019 | Monthly | 101.99 | 93.2 | 111.14 |
| Slovakia | SDR | Harmonic with Trend | 2015-2019 | Weekly | 135.22 | 125.44 | 145.13 |
| Slovakia | SDR | Harmonic with Trend | 2015-2019 | Monthly | 135.54 | 125.55 | 145.18 |
| Spain | CDR | Harmonic with Trend | 2015-2019 | Weekly | 173.3 | 170.53 | 176.16 |
| Spain | CDR | Harmonic with Trend | 2015-2019 | Monthly | 173.67 | 171.00 | 176.62 |
| Spain | SDR | Harmonic with Trend | 2015-2019 | Weekly | 151.98 | 149.22 | 154.6 |
| Spain | SDR | Harmonic with Trend | 2015-2019 | Monthly | 152.25 | 149.84 | 154.83 |
| Sweden | CDR | Harmonic with Trend | 2015-2019 | Weekly | 55.98 | 50.21 | 62.33 |
| Sweden | CDR | Harmonic with Trend | 2015-2019 | Monthly | 56.42 | 50.16 | 62.26 |
| Sweden | SDR | Harmonic with Trend | 2015-2019 | Weekly | 42.83 | 37.41 | 49.12 |
| Sweden | SDR | Harmonic with Trend | 2015-2019 | Monthly | 43.15 | 37.28 | 48.91 |
| Switzerland | CDR | Harmonic with Trend | 2015-2019 | Weekly | 115.81 | 109.57 | 122.00 |
| Switzerland | CDR | Harmonic with Trend | 2015-2019 | Monthly | 116.28 | 110.41 | 122.41 |
| Switzerland | SDR | Harmonic with Trend | 2015-2019 | Weekly | 108.8 | 103.02 | 115.00 |
| Switzerland | SDR | Harmonic with Trend | 2015-2019 | Monthly | 109.16 | 102.95 | 115.01 |
| Taiwan | CDR | Harmonic with Trend | 2015-2019 | Weekly | -40.83 | -44.6 | -37.11 |
| Taiwan | CDR | Harmonic with Trend | 2015-2019 | Monthly | -41.01 | -44.59 | -37.51 |
| Taiwan | SDR | Harmonic with Trend | 2015-2019 | Weekly | -54.51 | -58.61 | -50.53 |
| Taiwan | SDR | Harmonic with Trend | 2015-2019 | Monthly | -54.69 | -58.78 | -50.5 |
| U.S.A. | CDR | Harmonic with Trend | 2015-2019 | Weekly | 140.54 | 139.45 | 141.6 |
| U.S.A. | CDR | Harmonic with Trend | 2015-2019 | Monthly | 139.8 | 138.68 | 140.82 |
| U.S.A. | SDR | Harmonic with Trend | 2015-2019 | Weekly | 156.41 | 155.32 | 157.62 |
| U.S.A. | SDR | Harmonic with Trend | 2015-2019 | Monthly | 155.49 | 154.39 | 156.6 |

**Source**: Jdanov et al. (2021) and European Commission (2013).

**Table 4B:** Excess mortality rates (per 100,000 persons) and country ranking (in parentheses) for each scenario, 2020.

| **Country** | **Scenario**  **1** | **Scenario**  **2** | **Scenario**  **3** | **Scenario**  **4** | **Scenario**  **5** | **Scenario**  **6** | **Scenario**  **7** | **Scenario**  **8** | **Scenario**  **9** | **Scenario**  **10** | **Scenario**  **11** | **Scenario**  **12** |
| --- | --- | --- | --- | --- | --- | --- | --- | --- | --- | --- | --- | --- |
| **Lithuania** | 51.32 | 180.8 | 185.43 | 182.19 | 125.59 | 170.75 | 176.03 | 172.02 | 139.46 | 189.42 | 116.77 | 189.09 |
|  | (11) | (1) | (1) | (1) | (9) | (2) | (2) | (2) | (5) | (1) | (6) | (3) |
| **Poland** | 145.12 | 177.55 | 178.75 | 178.16 | 194.51 | 149.46 | 151.05 | 149.96 | 198.56 | 184.5 | 163.53 | 157.33 |
|  | (1) | (2) | (2) | (2) | (1) | (5) | (5) | (5) | (1) | (2) | (1) | (5) |
| **Belgium** | 90.69 | 159.32 | 159.38 | 159.08 | 142.28 | 169.25 | 169.52 | 169.02 | 142.29 | 170.89 | 150.96 | 183.37 |
|  | (4) | (3) | (3) | (3) | (7) | (3) | (4) | (3) | (4) | (3) | (2) | (4) |
| **U.S.A.** | 120.27 | 153.98 | 155.01 | 153.62 | 158.1 | 138.13 | 139.25 | 137.76 | - | 163.51 | - | 144.63 |
|  | (2) | (4) | (4) | (4) | (4) | (7) | (8) | (8) |  | (4) |  | (9) |
| **Spain** | 85.13 | 146.65 | 149.5 | 147.15 | 153.86 | 166.95 | 170.46 | 167.45 | 127.05 | 163.2 | 133.32 | 191.1 |
|  | (6) | (5) | (5) | (5) | (5) | (4) | (3) | (4) | (6) | (5) | (4) | (2) |
| **Hungary** | 69.34 | 146.43 | 149.25 | 146.72 | 145.16 | 144.21 | 147.27 | 144.34 | 145.84 | 149.74 | 143.18 | 148.65 |
|  | (8) | (6) | (6) | (6) | (6) | (6) | (6) | (6) | (2) | (10) | (3) | (8) |
| **Slovenia** | 92.99 | 142.23 | 144.12 | 141.91 | 159.6 | 134.01 | 136.37 | 134.06 | 145.07 | 157.57 | 132.67 | 153.56 |
|  | (3) | (7) | (7) | (7) | (3) | (9) | (9) | (9) | (3) | (6) | (5) | (7) |
| **Italy** | 89.44 | 140.37 | 143.55 | 141.63 | 194.41 | 185.06 | 189.54 | 186.62 | - | 155.19 | - | 204.38 |
|  | (5) | (8) | (8) | (8) | (2) | (1) | (1) | (1) |  | (8) |  | (1) |
| **England & Wales** | 78.98 | 138.94 | 141.8 | 140.39 | 113.45 | 137.19 | 140.11 | 138.68 | 109.34 | 156.9 | 109.8 | 154.56 |
|  | (7) | (9) | (9) | (9) | (10) | (8) | (7) | (7) | (8) | (7) | (7) | (6) |
| **Slovakia** | 28.75 | 131.02 | 133.39 | 133.05 | 97.84 | 98.00 | 100.4 | 99.43 | 125.32 | 154.25 | 97.78 | 109.95 |
|  | (15) | (10) | (10) | (10) | (12) | (13) | (13) | (13) | (7) | (9) | (11) | (13) |
| **Scotland** | 65.38 | 125.41 | 128.36 | 125.26 | 108.94 | 121.75 | 124.83 | 121.57 | 99.89 | 142.05 | 99.55 | 136.74 |
|  | (9) | (11) | (11) | (11) | (11) | (10) | (10) | (10) | (9) | (11) | (10) | (10) |
| **Switzerland** | 45.94 | 106.65 | 107.29 | 107.43 | 92.31 | 113.3 | 114.25 | 114.07 | 88.63 | 107.91 | 100.43 | 115.24 |
|  | (13) | (12) | (12) | (12) | (13) | (11) | (12) | (11) | (11) | (12) | (9) | (12) |
| **Portugal** | 55.15 | 89.67 | 94.55 | 91.19 | 131.3 | 110.36 | 116.22 | 112.07 | 95.41 | 95.37 | 101.78 | 122.33 |
|  | (10) | (13) | (13) | (13) | (8) | (12) | (11) | (12) | (10) | (13) | (8) | (11) |
| **Netherlands** | 48.45 | 85.96 | 86.68 | 85.62 | 86.62 | 77.5 | 78.39 | 77.19 | 78.2 | 92.86 | 65.18 | 84.1 |
|  | (12) | (14) | (14) | (14) | (15) | (15) | (15) | (15) | (12) | (14) | (13) | (15) |
| **France** | 31.68 | 74.89 | 76.63 | 75.49 | 90.9 | 83.48 | 85.83 | 84.1 | 66.52 | 85.29 | 72.77 | 99.56 |
|  | (14) | (15) | (15) | (15) | (14) | (14) | (14) | (14) | (13) | (15) | (12) | (14) |
| **Israel** | -4.59 | 65.68 | 67.74 | 66.55 | 14.57 | 37.9 | 39.15 | 38.27 | 41.58 | 68.86 | 24.03 | 40.51 |
|  | (17) | (16) | (16) | (16) | (20) | (18) | (18) | (18) | (15) | (16) | (16) | (19) |
| **Austria** | 20.74 | 60.6 | 62.18 | 61.83 | 62.85 | 68.45 | 70.17 | 69.73 | 54.91 | 64.18 | 57.91 | 70.16 |
|  | (16) | (17) | (17) | (17) | (16) | (16) | (16) | (16) | (14) | (18) | (14) | (17) |
| **Sweden** | -9.59 | 40.6 | 41.43 | 41.18 | 13.28 | 53.71 | 54.57 | 54.28 | 31.18 | 64.79 | 42.86 | 75.17 |
|  | (18) | (18) | (18) | (18) | (21) | (17) | (17) | (17) | (18) | (17) | (15) | (16) |
| **Estonia** | -38.14 | 24.87 | 27.68 | 24.35 | 23.51 | 17.49 | 21.04 | 17.01 | 39.27 | 36.01 | 20.18 | 37.69 |
|  | (19) | (19) | (19) | (19) | (19) | (19) | (19) | (19) | (16) | (20) | (17) | (20) |
| **Latvia** | -40.73 | 20.18 | 22.00 | 19.67 | 27.83 | 10.79 | 13.12 | 10.02 | 31.72 | 53.51 | 7.48 | 49.19 |
|  | (20) | (20) | (20) | (20) | (18) | (20) | (20) | (20) | (17) | (19) | (21) | (18) |
| **R.of Korea** | -70.9 | 10.57 | 11.09 | 10.3 | 29.94 | 7.77 | 8.7 | 7.54 | 12.77 | 26.73 | 10.12 | 16.51 |
|  | (24) | (21) | (21) | (21) | (17) | (21) | (21) | (21) | (19) | (21) | (20) | (21) |
| **Finland** | -47.86 | -5.32 | -3.14 | -4.09 | 12.05 | -3.14 | -0.59 | -1.96 | -3.22 | 5.17 | -1.4 | 7.51 |
|  | (22) | (22) | (22) | (22) | (22) | (22) | (22) | (22) | (22) | (22) | (22) | (22) |
| **Norway** | -54.34 | -9.88 | -8.26 | -8.72 | -19.57 | -3.33 | -1.85 | -2.39 | -2.17 | -6.29 | 10.94 | -1.56 |
|  | (23) | (23) | (23) | (23) | (25) | (23) | (23) | (23) | (21) | (23) | (19) | (23) |
| **Denmark** | -42.3 | -12.42 | -13.28 | -11.55 | 5.83 | -5.1 | -5.7 | -4.36 | 12.42 | -8.37 | 16.23 | -2.46 |
|  | (21) | (24) | (24) | (24) | (23) | (24) | (24) | (24) | (20) | (24) | (18) | (24) |
| **Taiwan** | -108.45 | -53.45 | -54.51 | -53.45 | -13.85 | -40.75 | -40.83 | -40.75 | -65.38 | -41.54 | -50.31 | -34.96 |
|  | (26) | (25) | (25) | (25) | (24) | (25) | (25) | (25) | (23) | (25) | (23) | (26) |
| **New Zealand** | -82.95 | -56.6 | -57.24 | -55.32 | -33.65 | -42.73 | -42.92 | -41.68 | - | -44.76 | - | -33.84 |
|  | (25) | (26) | (26) | (26) | (26) | (26) | (26) | (26) |  | (26) |  | (25) |

**Notes**: Table is ordered by the Scenario 2. See Table 1 for more details about the scenarios.

**Source**: Author’s elaboration.

**Appendix C**

*Supplementary Figure*

**Figure 1C –** Differences between excess mortality rates by using monthly instead of weekly data for each mortality index and country, 2020


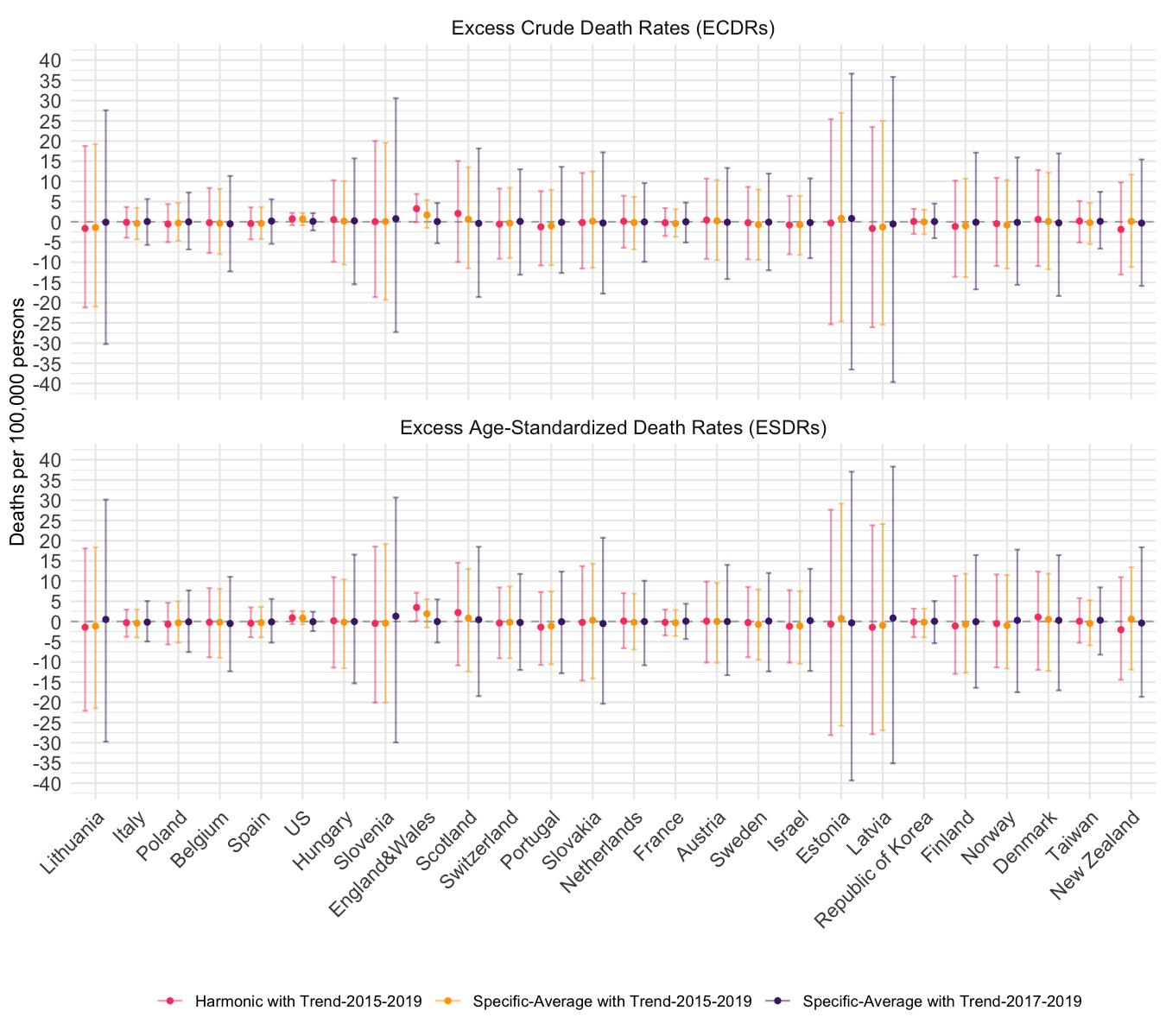


*Notes:* The differences are computed as, excess mortality derived from the weekly death series minus excess mortality derived from the monthly death series.

95% confidence intervals are based on Monte Carlo simulation.

***Sources:*** Jdanov et al. (2021) and European Commission (2013)**.**
